# IRF8 Drives Conventional Type 1 Dendritic Cell Differentiation and CD8^+^ T Cell Activation to Aggravate Abdominal Aortic Aneurysm Development

**DOI:** 10.1002/advs.202416238

**Published:** 2025-04-04

**Authors:** Zhen Yuan, Li Shu, Yidan Zheng, Yidong Wang, Mengsha Zheng, Jie Sun, Jiantao Fu, Zihao Zhou, Shen Song, Zhenjie Liu, Fei Li, Zhejun Cai

**Affiliations:** ^1^ Department of Cardiology The Second Affiliated Hospital, School of Medicine Heart Regeneration and Repair Key Laboratory of Zhejiang Province Hangzhou 310009 China; ^2^ State Key Laboratory of Transvascular Implantation Devices Hangzhou 310009 China; ^3^ Heart Regeneration and Repair Key Laboratory of Zhejiang Province Hangzhou 310009 China; ^4^ Transvascular Implantation Devices Research Institute Hangzhou 310053 China; ^5^ Department of Cardiovascular Surgery Union Hospital Tongji Medical College Huazhong University of Science and Technology Wuhan 430022 China; ^6^ Department of Pathology The Second Affiliated Hospital Zhejiang University School of Medicine Hangzhou 310009 China; ^7^ Beijing Anzhen Hospital of Capital Medical University and Beijing Institute of Heart Lung and Blood Vessel Diseases Beijing 100029 China; ^8^ Clinical Center for HIV/AIDS Beijing Ditan Hospital Capital Medical University Beijing 100015 China; ^9^ State Key Laboratory of Cardiovascular Disease Fuwai Hospital National Center for Cardiovascular Disease Chinese Academy of Medical Sciences and Peking Union Medical College Beijing 100037 China; ^10^ Department of Vascular Surgery The Second Affiliated Hospital Zhejiang University School of Medicine Hangzhou 310009 China; ^11^ Department of Structural Heart Disease Fuwai Yunnan Cardiovascular Hospital Kunming 650102 China

**Keywords:** abdominal aortic aneurysm, CD8^+^ T cell, dendritic cell, IRF8

## Abstract

Abdominal aortic aneurysm (AAA) is the most common true aneurysm worldwide, and recent studies suggest that dendritic cells (DCs) play a key role in its development, though the specific subtypes and underlying mechanisms remain unclear. In this study, the role of interferon regulatory factor 8 (IRF8) in AAA is investigated by focusing on its effect on the differentiation of DC precursors into conventional type 1 dendritic cells (cDC1s). It is found significant infiltration of HLA‐DR^+^ IRF8^+^ cells in human AAA tissue samples. In mice, DC‐specific overexpression of Irf8 exacerbates aneurysm expansion following periadventitial elastase application, while DC‐specific *Irf8* deletion attenuates AAA development. *Batf3* ^−/−^ mice, which lack cDC1s, exhibit AAA characteristics similar to the *Irf8*‐deleted mice. Additionally, an increased population of activated CD8^+^ T cells is observed in the DC‐*Irf8* overexpressed mice, while the DC‐*Irf8* deletion mice show a decrease in these cells. Blocking antigen cross‐presentation to CD8^+^ T cells also reduces AAA progression. Tissue microarray analysis of human aortic samples further confirms a correlation between IRF8 expression and AAA development. These findings suggest that IRF8 activation promotes cDC1 differentiation, leading to the recruitment of CD8^+^ T cells, which contribute to aortic wall destruction and AAA formation.

## Graphical abstract

This study highlights the critical role of IRF8 in the development of AAA. IRF8 activation promotes the differentiation of cDC1s, which in turn recruit and activate CD8+ T cells, contributing to aortic wall degradation. The study identifies the IRF8‐cDC1‐CD8+ T cell axis as a key pathway in AAA progression, offering new potential therapeutic targets to inhibit disease progression.

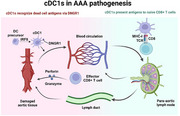

## 1 Introduction

Abdominal aortic aneurysm (AAA) is characterized by a permanent and localized expansion of the infrarenal segment of the aorta, often presenting asymptomatically or with nonspecific abdominal discomfort.^[^ ^1^ ^]^ In developed countries, the prevalence of AAA ranges from ≈4% to 8%, with a significantly higher incidence in males than in females.^[^ ^2^ ^]^ The most critical and life‐threatening complication of AAA is aneurysm rupture, primarily resulting from the progressive increase in aortic diameter and wall stress. Current treatment options include endovascular aneurysm repair and urgent surgical intervention, recommended for patients with significant enlargement (diameter greater than 55 mm), symptomatic aneurysms, or ruptured aneurysms.^[^ ^3^ ^]^ However, effective pharmaceutical treatments are lacking for patients who do not meet surgical criteria.

AAA is characterized by several pathological changes, including proteolytic fragmentation of the extracellular matrix (ECM), vascular smooth muscle cell (VSMC) death, immune cell infiltration into the adventitia, and increased oxidative stress within the aortic wall.^[^ ^4^ ^]^ Collectively, these alterations contribute to the weakening and subsequent dilation of the aortic structure.^[^ ^5^ ^]^ Immune responses are increasingly recognized as pivotal in AAA pathogenesis, highlighting the need for novel therapeutic strategies, especially for asymptomatic patients.^[^ ^6^ ^]^ Growing evidence underscores the importance of macrophage‐related mechanisms, such as proinflammatory M1‐like polarization and NLRP3 inflammasome.^[^ ^7^ ^]^ Recent studies have also highlighted the critical role of other immune cells, including T cells and neutrophils, in AAA development.^[^ ^8^ ^]^ These cells contribute to chronic inflammation and vascular remodeling, further promoting disease progression.

Dendritic cells (DCs), as professional antigen‐presenting cells, consist of diverse subsets and play a critical role in immune responses by continuously processing and presenting antigens—a function that is significantly enhanced upon activation.^[^ ^9^ ^]^ Previous studies have shown that depletion of CD11c^+^ DCs significantly attenuates AAA expansion in murine models.^[^ ^10^ ^]^ Nevertheless, the precise roles and mechanisms of DCs in AAA pathogenesis remain insufficiently explored. Dendritic cells encompass various subtypes, each with distinct roles and characteristics. Our previous single‐cell sequencing analysis of elastase‐induced AAA samples revealed that the conventional type 1 dendritic cell (cDC1) subpopulation exhibited the most significant infiltration among DC subtypes. This finding suggests a potentially crucial role for cDC1 cells in AAA development or progression, underscoring the need for further investigation.

Interferon regulatory factor 8 (IRF8) is vital for DC development, particularly within the cDC1 lineage.^[^ ^11^ ^]^ IRF8 is essential for the differentiation of macrophage and DC precursors into common dendritic cell precursors (CDPs) and for the subsequent transition of pre‐cDC1s from CDPs.^[^ ^12^ ^]^ Maturation into the cDC1 lineage is further facilitated by BATF3‐dependent IRF8 autoactivation.^[^ ^13^ ^]^ Additionally, IRF8 expression in mature cDC1 cells is crucial for their survival, highlighting its significance at multiple stages of cDC1 development.^[^ ^14^ ^]^ Previous research has shown that targeted deletion of IRF8 in DCs leads to reduced accumulation of aortic CD103^+^CD11b^−^ DCs and significantly diminishes the development of atherosclerosis.^[^ ^15^ ^]^ These observations suggest that IRF8 expression may influence AAA development by regulating cDC1 differentiation.

In this study, we investigate the role of IRF8 in AAA. We found that IRF8 in DCs contributes to AAA development by promoting cDC1 maturation. Our findings indicate that cDC1s promote the presentation of dead‐cell antigens to CD8^+^ T cells, leading to apoptosis of smooth muscle cells (SMCs) in the abdominal aorta. Corroborating evidence from human samples further confirmed the involvement of IRF8 in AAA pathogenesis.

## 2 Result

### 2.1 IRF8 is Upregulated in Human and Murine AAA Samples

**Figure 1:**
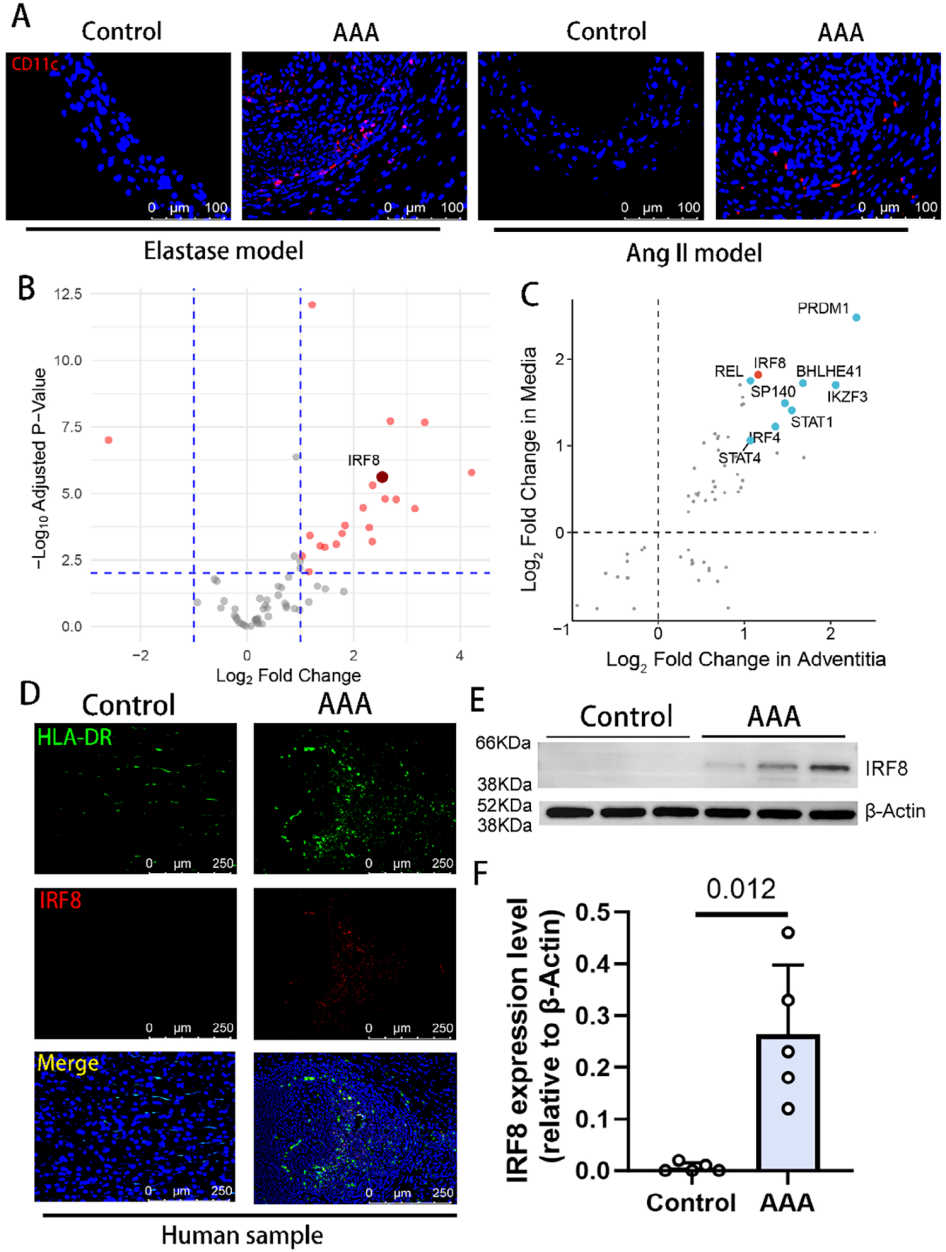
IRF8 is upregulated in human and murine AAA samples. A) Representative immunofluorescence images of C57BL6/J aortic sections 14 days after peri‐adventitial elastase application model and *Apoe* ^−/−^ aortic sections 28 days after Angiotensin‐II mini‐pump implantation, with staining for CD11c (red), and nuclei (blue) (*n* = 4 of each group). Scale bar, 100 µm. B) Volcano plot of the differentially expressed transcription factors related to immune responses (GO:0 006955) in the GSE183464 dataset, The deep red dot represents IRF8. The vertical dashed line means log fold change = 1 and the horizontal dashed line means *P* = 0.01. C) Upregulated transcription factors in the tunica adventitia and tunica media of human AAAs versus healthy aortas. Data was extracted from an expression profiling by the array (GSE232911). D) Representative images of immunofluorescence imaging for the IRF8 and dendritic cell marker HLA‐DR in aortic tissues of human healthy aortas or AAA samples with staining for HLA‐DR (green), IRF8 (red) and nuclei (blue) (*n* = 5 of each group). Scale bar, 250 µm. E) Representative bands of IRF8 protein from the aortas of C57BL6/J mice subjected to sham operation or peri‐adventitial elastase application 14 days after surgery. F) Quantification of the IRF8 expression level of aortic samples (relative to β‐Actin) 14 days after the application of peri‐adventitial elastase or vehicle (*n* = 5 of each group, unpaired two‐tailed Student's *t*‐test). IRF8, interferon regulatory factor 8; AAA, abdominal aortic aneurysm; DC, dendritic cell; KEGG, Kyoto Encyclopedia of Genes and Genomes.

Initially, we observed an increased population of CD11c^+^ cells within the intimal and adventitial layers of mice 14 days after elastase induction (**Figure** **1**A). Similarly, *Apoe* ^−/−^ mice exhibited enhanced infiltration of CD11c^+^ cells after 28 days of continuous angiotensin II (Ang II) infusion (Figure 1A). To translate these findings to human disease, we analyzed transcriptome sequencing data from male AAA patient tissues (GSE183464 dataset). Kyoto Encyclopedia of Genes and Genomes (KEGG) pathway analyses indicated that the upregulated genes were enriched in pathways related to inflammation and cell adhesion (Figure S1A, Supporting Information). Focusing on upregulated transcription factors associated with the Gene Ontology (GO) term “immune response” (GO:0 006955), we identified IRF8 as one of the most significantly altered genes (Figure 1B). Further supporting this observation, microarray expression profiling of 246 media and adventitia samples from aortic tissues revealed that IRF8 was significantly upregulated among transcription factors in the AAA group (Figure 1C).^[^ ^16^ ^]^ ImmGen analysis of these differentially expressed transcription factors confirmed that IRF8 is highly enriched in the dendritic cell lineage (Figure S1B, Supporting Information),^[^ ^17^ ^]^ suggesting that IRF8 may mediate the pro‐expansion effects of dendritic cells in AAA. Validating these observations in human aortic tissues, examination of paraffin‐embedded sections from AAA patients demonstrated a higher infiltration of IRF8^+^ HLA‐DR^+^ cells compared to healthy donors (Figure 1D). Consistently, AAA tissues from elastase‐induced mice displayed elevated IRF8 expression levels compared to controls (Figure 1E,F). Collectively, these findings implicate dendritic cell‐derived IRF8 in the pathogenesis of AAA.

### 2.2 IRF8 Expression Regulates Murine AAA Development

**Figure 2:**
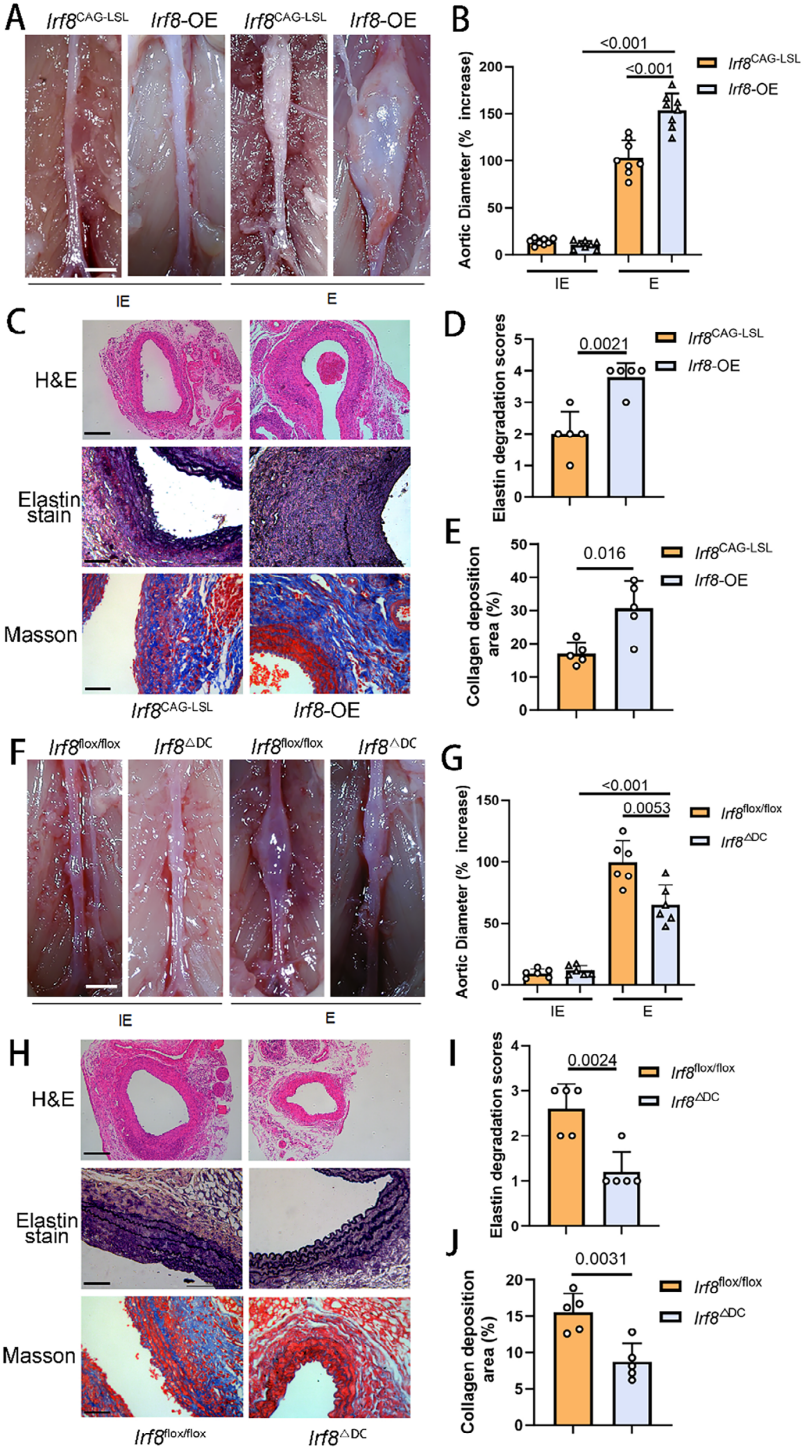
IRF8 expression level regulates murine AAA development. A) Representative images of the gross anatomy of murine abdominal aortas 14 days after the application of vehicle (inactive elastase, IE) or peri‐adventitial elastase (marked by E in the figure) in the control and *Irf8*‐OE groups (*n* = 8 of each group). Scale bar, 1 mm. B) Quantification of murine AAA expansion rate 14 days after the application of vehicle or peri‐adventitial elastase in the control and *Irf8*‐OE groups (*n* = 8 of each group, two‐way ANOVA using Tukey's multiple comparisons test). C) Representative images of histology of abdominal aortas of the control and *Irf8*‐OE groups 14 days after application of peri‐adventitial elastase. Scale bar: 200 µm for H&E staining and 50 µm for elastin Van Gieson and Masson staining. D) Quantification of the elastin degradation score of AAA sections 14 days after the application of peri‐adventitial elastase in the control and *Irf8*‐OE groups (*n* = 5 of each group, unpaired two‐tailed Student's *t*‐test). E) Quantification of the collagen deposition area of AAA sections using Masson staining 14 days after the application of peri‐adventitial elastase in the control and *Irf8*‐OE groups (*n* = 5 of each group, unpaired two‐tailed Student's *t*‐test). F) Representative images of the gross anatomy of murine abdominal aortas 14 days after the application of vehicle or peri‐adventitial elastase in the control and *Irf8* ^ΔDC^ groups (*n* = 6 of each group). Scale bar, 1 mm. G) Quantification of murine AAA expansion rate 14 days after the application of vehicle or peri‐adventitial elastase in the control and *Irf8* ^ΔDC^ groups (*n* = 6 of each group, two‐way ANOVA using Tukey's multiple comparisons test). H) Representative images of histology of abdominal aortas of the control and *Irf8* ^ΔDC^ groups 14 days after application of peri‐adventitial elastase. Scale bar: 200 µm for H&E staining and 50 µm for elastin Van Gieson staining. I) Quantification of the elastin degradation score of AAA sections 14 days after the application of peri‐adventitial elastase in the control and *Irf8* ^ΔDC^ groups (*n* = 5 of each group, unpaired two‐tailed Student's *t*‐test). J) Quantification of the collagen deposition area of AAA sections using Masson staining 14 days after the application of peri‐adventitial elastase in the control and *Irf8*‐OE groups (*n* = 5 of each group, unpaired two‐tailed Student's *t*‐test). IRF8, interferon regulatory factor 8; AAA, abdominal aortic aneurysm; OE, overexpression; ANOVA, Analysis of Variance.

Given the elevated IRF8 expression observed in both human and murine AAA samples, we investigated the role of IRF8 activation in AAA expansion by developing various murine models with distinct genotypes, all induced with periadventitial elastase. We first utilized *Irf8* ^CAG‐LSL^ *Itgax* ^Cre^ mice (overexpression group, hereafter referred to as *Irf8*‐OE), which showed significantly more severe AAA expansions compared to controls (**Figure** **2**A,B). Pathological examinations revealed that the *Irf8*‐OE group had more dilated aortic lumens, disrupted multilayer elastin structures compared to the healthy abdominal aortas (Figure S2A, Supporting Information), and increased collagen deposition (Figure 2C–E). Moreover, AAA sections from *Irf8*‐OE mice showed increased infiltration of inflammatory cells, including macrophages and neutrophils (Figure S2B, Supporting Information). We also examined *Irf8* ^flox/flox^ *Itgax* ^Cre^ mice (conditional *Irf8* knockout in dendritic cells, hereafter *Irf8* ^ΔDC^), finding that IRF8 downregulation substantially reduced AAA expansion rates (Figure 2F,G). Furthermore, these mice exhibited minimal pathological changes and were largely protected from inflammatory cell infiltration and collagen deposition in their abdominal aortas (Figure 2H–J; and Figure S2C, Supporting Information). Our results suggest that IRF8 expression significantly influences AAA expansion following elastase application.

### 2.3 IRF8 is Dominantly Expressed by cDC1s in AAA Microenvironment

**Figure 3:**
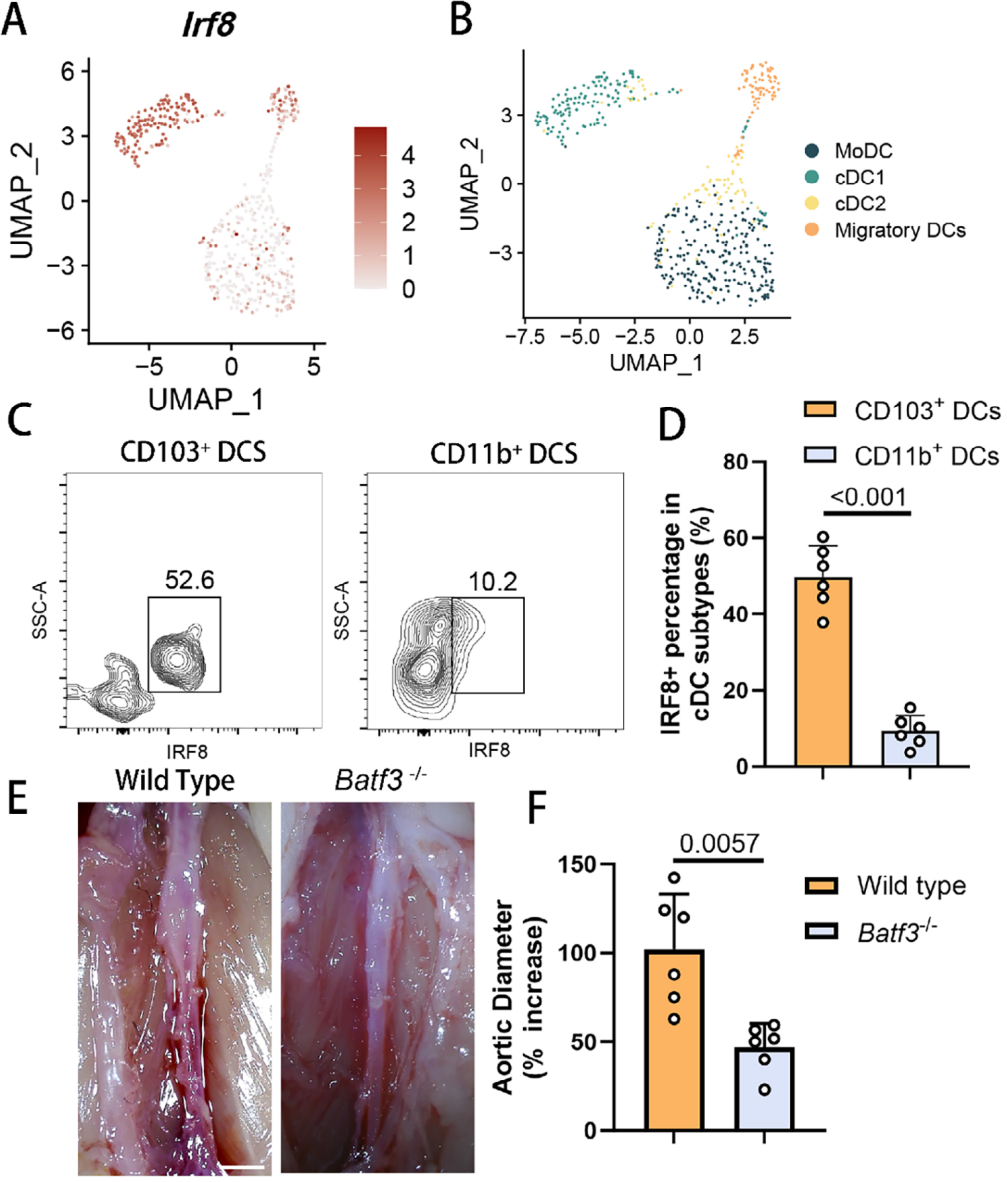
IRF8 is dominantly expressed by cDC1s in AAA microenvironment. A) UMAP projection of the expression profile of IRF8 in the subtypes of dendritic cells in murine AAA samples at day 0, day 7, and day 14 postsurgery. Each point depicts a single cell, colored according to normalized expression levels. B) UMAP visualization of the 4 subclusters of dendritic cells in the murine AAA samples. C) Representative images for flow cytometry analysis of IRF8 expression in dendritic cells from aortic tissues of elastase‐induced C57BL6/J male mice. Live cells were gated, doublets were excluded, and CD45^+^ cells were identified. MHC II^+^ CD11c^+^ CD103^+^ CD11b^−^ cells were identified as cDC1s. MHC II^+^ CD11c^+^ CD11b^+^ CD103^−^ cells were identified as cDC2s. D) Flow cytometry‐based quantification of IRF8 expression in cDC1s and cDC2s (*n* = 6 of each group, unpaired two‐tailed Student's *t*‐test). E) Representative images of the gross anatomy of murine abdominal aortas 14 days after the application of peri‐adventitial elastase in the control and *Batf3* ^−/−^ groups (*n* = 6 of each group). Scale bar, 1 mm. F) Quantification of murine AAA expansion rate 14 days after the application of peri‐adventitial elastase in the control and *Batf3* ^−/−^ groups (*n* = 6 of each group, unpaired two‐tailed Student's *t*‐test). cDC1, conventional type 1 dendritic cell; AAA, abdominal aortic aneurysm; MHC, major histocompatibility complex; IRF8, interferon regulatory factor 8.

We analyzed single‐cell transcriptomic sequencing data from elastase‐induced murine AAA models. Among the myeloid lineage of DC subtypes, the cDC1 population expanded dramatically during AAA development (Figure S3A, Supporting Information) and was the primary cell type expressing IRF8 (**Figure** **3**A,B). To confirm this observation, we performed flow cytometry on the DC population, uncovering that the majority of IRF8^+^ cells were CD103^+^ CD11b^−^ cDC1s (Figure 3C,D; and Figure S3B, Supporting Information). To elucidate the role of IRF8‐expressing cDC1s in the AAA microenvironment, we utilized *Batf3* ^−/−^ mice, which lack cDC1s throughout the body.^[^ ^18^ ^]^ These mice exhibited phenotypes similar to those observed in models with *Irf8* depletion (Figure 3E,F; and Figure S3C, Supporting Information), suggesting that cDC1 acts as the cellular mediator of IRF8 function during AAA development.

### 2.4 IRF8‐Stimulated cDC1 Cells Activate CD8^+^ T Cell Maturation

**Figure 4:**
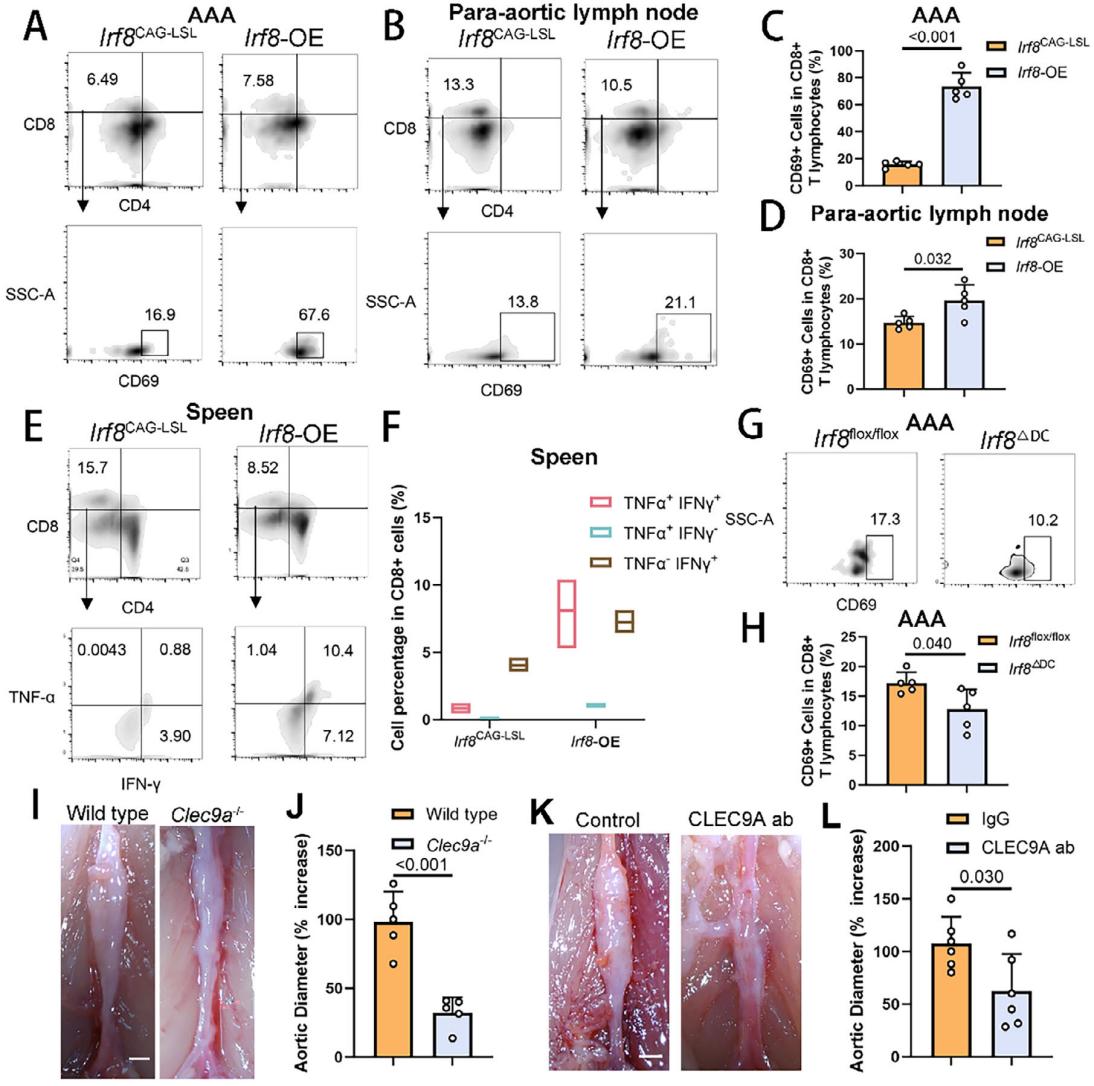
IRF8‐Stimulated cDC1 Cells Activate CD8+ T Cell Maturation. A) Representative images for flow cytometry analysis of T cell presence in aortic tissues in control or *Irf8*‐OE male mice. Live cells were gated, doublets were excluded, and CD45^+^ cells were identified. CD3e^+^ CD4^+^ cells were identified as CD4^+^ T cells. CD3e^+^ CD8^+^ cells were identified as CD8^+^ T cells. CD69 expression reflects the percentage of mature T cells. B) Representative images for flow cytometry analysis of T cell presence in para‐aortic lymph nodes in control or *Irf8*‐OE male mice. C) Flow cytometry‐based quantification of the percentage of CD69^+^ cells in CD8+ T cells within aortic tissues in control or *Irf8*‐OE male mice (*n* = 5 of each group, unpaired two‐tailed Student's *t*‐test). D) Flow cytometry‐based quantification of the percentage of CD69^+^ cells in CD8^+^ T cells within para‐aortic lymph nodes in control or *Irf8*‐OE male mice (*n* = 5 of each group, unpaired two‐tailed Student's *t*‐test). E) Representative images for flow cytometry analysis of T cell presence in spleens in control or *Irf8*‐OE male mice. TNF‐α and IFN‐γ expression reflects the percentage of activated CD8^+^ T cells. F) Flow cytometry‐based quantification of the expression level of TNF‐α and IFN‐γ in CD8^+^ T cells within spleens in control or *Irf8*‐OE male mice (*n* = 5 of each group). G) Representative images for flow cytometry analysis of mature CD8^+^ T cell percentage in aortic tissues in control or *Irf8* ^ΔDC^ male mice. H) Flow cytometry‐based quantification of the percentage of CD69^+^ cells in CD8^+^ T cells within aortic tissues in control or *Irf8* ^ΔDC^ male mice (*n* = 5 of each group, unpaired two‐tailed Student's *t*‐test). I) Representative images of the gross anatomy of murine abdominal aortas 14 days after the application of peri‐adventitial elastase in the control and *Clec9a* ^−/−^ groups (*n* = 5 of each group). Scale bar, 1 mm. J) Quantification of murine AAA expansion rate 14 days after the application of peri‐adventitial elastase in the control and *Clec9a* ^−/−^ groups (*n* = 5 of each group, two‐way ANOVA using Tukey's multiple comparisons test). K) Representative images of the gross anatomy of murine abdominal aortas 14 days after the application of peri‐adventitial elastase in C57BL6/J male mice i.p. injected with control IgG or CLEC9A neutralizing antibody. The injections were implemented every other day at 400 µg per dose from the surgery day (*n* = 5 of each group). Scale bar, 1 mm. L) Quantification of murine AAA expansion rate 14 days after the application of peri‐adventitial elastase in C57BL6/J male mice i.p. injected with control IgG or CLEC9A neutralizing antibody (*n* = 5 of each group, two‐way ANOVA using Tukey's multiple comparisons test). DC, dendritic cell; AAA, abdominal aortic aneurysm; OE, overexpression; i.p., intraperitoneal.

Given that the primary role of cDC1s in immune responses is to present antigens to CD8^+^ T cells, we further investigated the involvement of CD8^+^ T cells in AAA progression. We observed a significant increase in CD8^+^ T cells expressing CD69—a well‐recognized early‐stage marker for T cell maturation—within the aneurysms and para‐aortic lymph nodes of *Irf8*‐OE mice (**Figure** **4**A–D; and Figure S4A, Supporting Information). Additionally, CD8^+^ T cells in the spleens of these *Irf8*‐OE mice exhibited elevated levels of TNF‐α and IFN‐γ (Figure 4E,F; and Figure S4B, Supporting Information), reflecting systemic activation of cytotoxicity associated with high IRF8 expression. In contrast, a marked decrease in CD69^+^ mature CD8^+^ T cells was observed in *Irf8* ^ΔDC^ mice (Figure 4G,H). To further delineate the role of CD8^+^ T cells in AAA pathogenesis, we employed *Clec9a* ^−/−^ mice, which are deficient in cDC1–CD8^+^ T cell communication.^[^ ^19^ ^]^ Remarkably, these mice exhibited a reduction in aneurysm expansion similar to that observed in *Irf8* ^ΔDC^ mice (Figure 4I,J). In addition, intraperitoneal administration of CLEC9A‐neutralizing antibodies effectively mitigated elastase‐induced AAA dilation compared with those receiving control IgG (Figure 4K,L). These findings highlight the significant roles of IRF8‐expressing cDC1s and CD8^+^ T cells in the progression of AAA.

### 2.5 Aortic Structures in the *Irf8*‐OE Group Suffered Increased Damage and Cell Apoptosis

**Figure 5:**
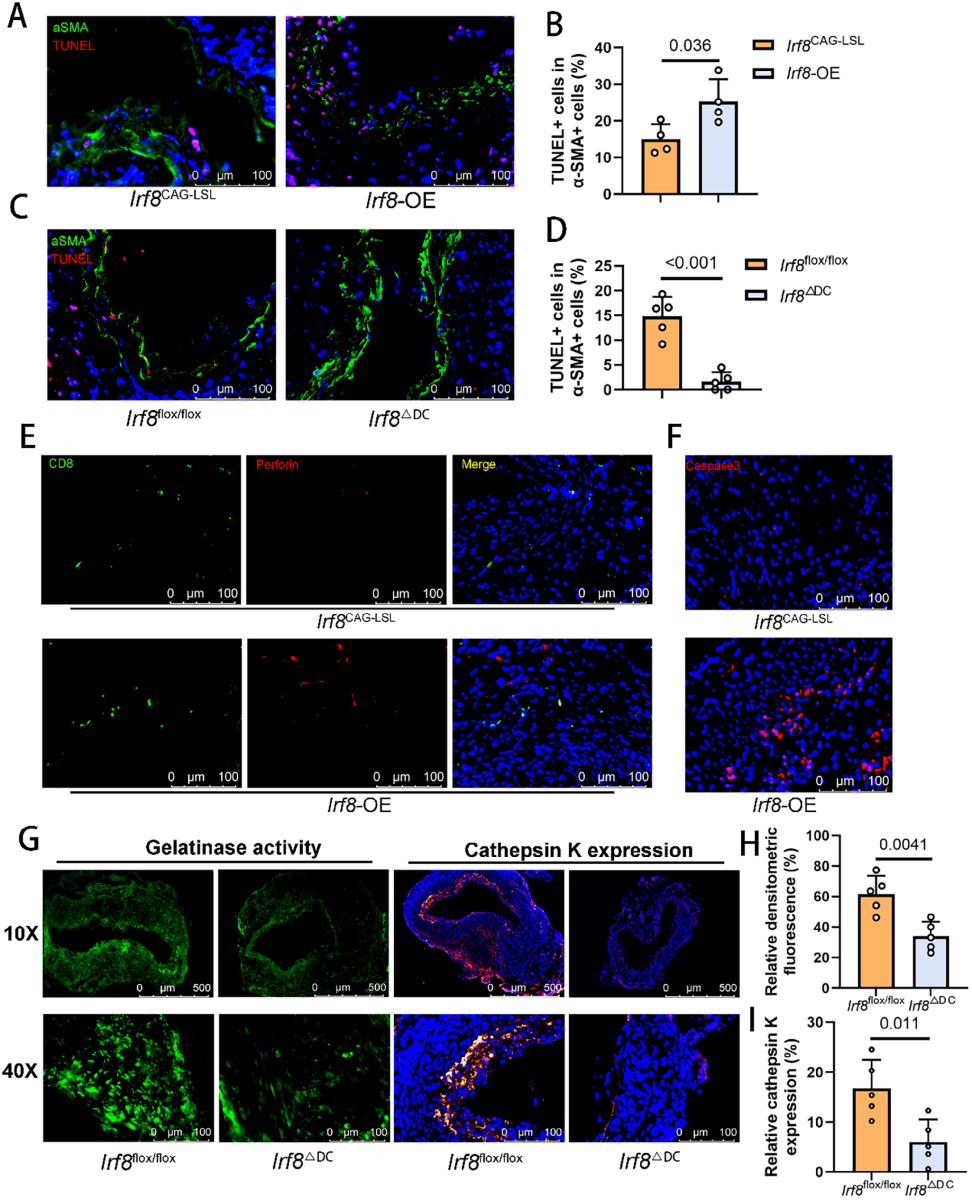
Aortic structure in the *Irf8*‐OE group suffered increased damage and cell apoptosis. A) Representative immunofluorescence images of aortic sections 14 days after peri‐adventitial elastase application in control or *Irf8*‐OE male mice with staining for TUNEL apoptosis detection kit (red), α‐SMA (green) and nuclei (blue) (*n* = 4 of each group). Scale bar, 100 µm. B) Quantification of the percentage of TUNEL^+^ cell percentage in α‐SMA^+^ cells within aortic tissues in control or *Irf8*‐OE male mice (*n* = 4 of each group, unpaired two‐tailed Student's *t*‐test). C) Representative immunofluorescence images of aortic sections 14 days after peri‐adventitial elastase application in control or *Irf8* ^ΔDC^ male mice with staining for TUNEL apoptosis detection kit (red), α‐SMA (green) and nuclei (blue) (*n* = 5 of each group). Scale bar, 100 µm. D) Quantification of the percentage of TUNEL^+^ cell percentage in α‐SMA^+^ cells within aortic tissues in control or *Irf8* ^ΔDC^ male mice (*n* = 5 of each group, unpaired two‐tailed Student's *t*‐test). E) Representative immunofluorescence images of aortic sections from the control and *Irf8*‐OE group 14 days after peri‐adventitial elastase application with staining for CD8 (green), perforin (red) and nuclei (blue) (*n* = 5 of each group). Scale bar, 100 µm. F) Representative immunofluorescence images of aortic sections from the control and *Irf8*‐OE group 14 days after peri‐adventitial elastase application with staining for Caspase3 (red) and nuclei (blue) (*n* = 4 of each group). Scale bar, 100 µm. G) Representative images of in situ zymography (left) and immunofluorescence of cathepsin K from the control or *Irf8* ^ΔDC^ male mice 14 days after peri‐adventitial elastase application. Scale bar, 500 µm for 10X images and 100 µm for 40X images. H) Quantification of the relative densitometric fluorescence identifying the gelatinase activity within aortic tissues in control or *Irf8* ^ΔDC^ male mice by in situ zymography (*n* = 5 of each group, unpaired two‐tailed Student's *t*‐test). I) Quantification of the relative expression level of cathepsin K within aortic tissues in control or *Irf8* ^ΔDC^ male mice with immunofluorescence (*n* = 5 of each group, unpaired two‐tailed Student's *t*‐test). OE, overexpression; TUNEL, Terminal deoxynucleotidyl transferase dUTP Nick‐End Labeling; SMA, smooth muscle cell.

CD8+ T cells mediate their primary effects through cytotoxicity. Utilizing a TUNEL assay, we demonstrated varying degrees of apoptosis in SMCs across different mouse genotypes, with the *Irf8*‐OE group exhibiting markedly more pronounced tissue destruction (**Figure** **5**A,B). In contrast, the aortic structure in Irf8ΔDC mice was largely preserved, with minimal SMC apoptosis (Figure 5C,D). Furthermore, cytotoxic CD8^+^ T cells significantly infiltrated in the AAA sections of the *Irf8*‐OE group, with extensive targeted cells showing perforin binding on their membranes (Figure 5E), while CD8 and perforin expressions were lower in the *Irf8* ^ΔDC^ group (Figure S5A, Supporting Information). Additionally, immunofluorescence analysis using a caspase‐3 antibody further confirmed a higher degree of apoptosis in the AAA tissues of *Irf8*‐OE mice (Figure 5F). Subsequently, we performed in situ gelatin zymography combined with fluorescence imaging to assess matrix metalloproteinases (MMPs) 2/9 activity and cathepsin K distribution in aortic sections from AAA induced by periadventitial elastase in mice of distinct genotypes (Figure 5G). The results revealed reduced gelatinase activity and decreased cathepsin K expression in the *Irf8* ^ΔDC^ group (Figure 5H,I), whereas no significant differences were observed in *Irf8*‐OE mice compared to controls (Figure S5B, Supporting Information), likely because cDC1 and T cells are not the main gelatinase producers. Collectively, these findings suggest that CD8^+^ T cells, activated by cDC1s, play a pivotal role in driving aortic degradation.

### 2.6 In Vitro Studies Identified Distinct Properties Affected by IRF8 Expression

**Figure 6:**
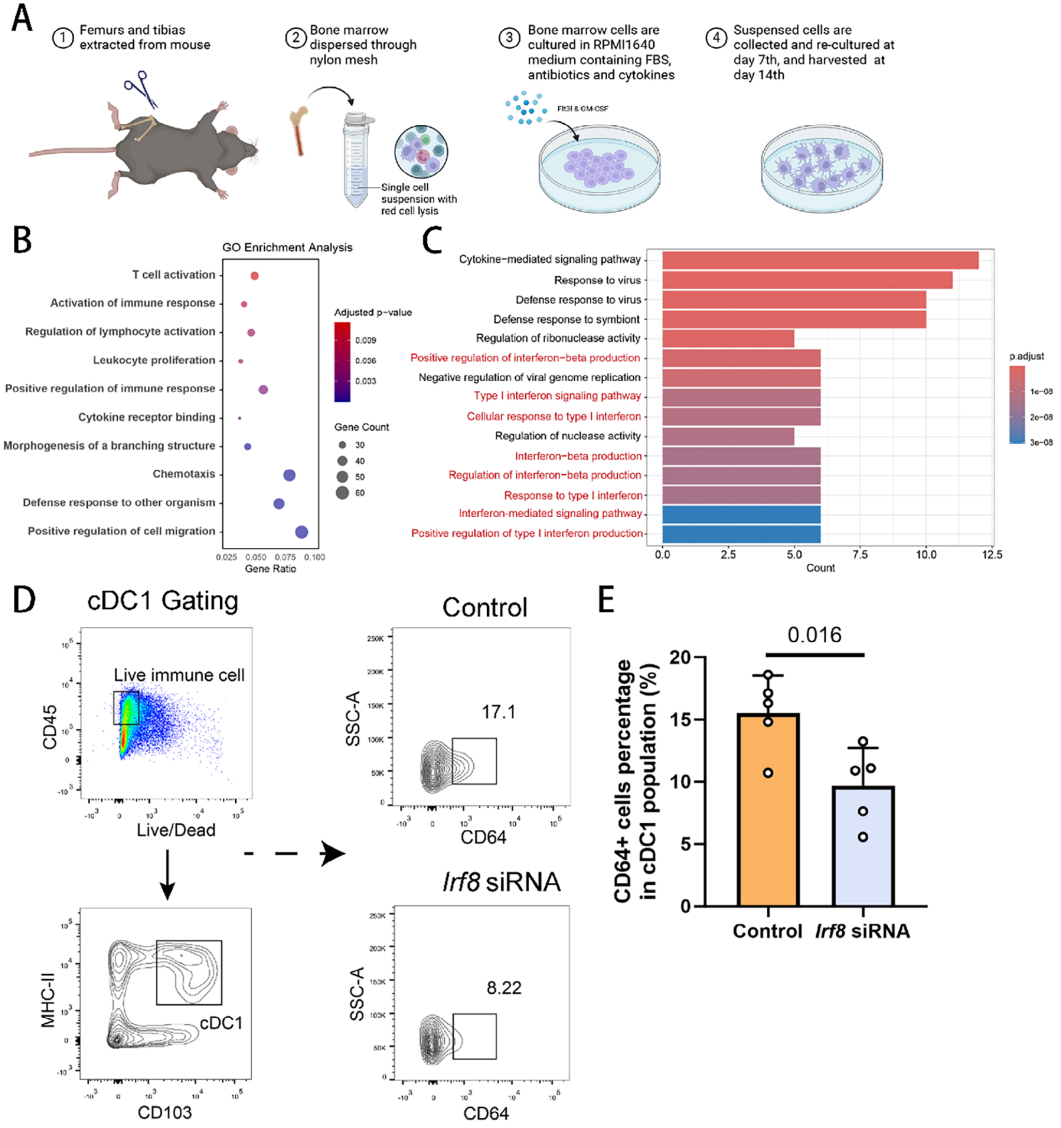
In vitro studies identified distinct properties affected by IRF8 expression. A) Graphical illustration of the experimental setup for bone‐marrow derived CD103^+^ DC cultivation. B) Dot plot of GO enrichment analyses with terms of interest for the transcriptional level of upregulated genes of *Irf8*‐OE BMDCs versus control ones. C) Dot plot of GO enrichment analyses exhibiting the top 15 enriched terms for the upregulated genes related to IRF8 of *Irf8*‐OE BMDCs. D) Gating strategy and representative images for flow cytometry analysis of CD64 expression in cDC1s from BMDCs either treated with control RNA or *Irf8* siRNA before stimulated by TNF‐α. Live cells were gated, doublets were excluded, and CD45^+^ cells were identified. MHC II^+^ CD103^+^ cells were identified as cDC1s. E) Flow cytometry‐based quantification of CD64 expression in BMDCs either treated with control RNA or *Irf8* siRNA (*n* = 5 of each group, unpaired two‐tailed Student's *t*‐test). IRF8, interferon regulatory factor 8; DC, dendritic cell; GO, gene ontology; *Irf8*‐OE, overexpression.

Our findings suggest that IRF8 expression levels are upregulated in both human and murine AAA samples, potentially accelerating aneurysm development by promoting the differentiation of cDC1s and the activation of CD8^+^ T cells. To further explore the biological functions of IRF8 in the inflammatory microenvironment of AAA tissue, we isolated primary bone marrow cells from the tibias and femurs of 8‐week‐old male mice and cultured them in RPMI 1640 medium supplemented with GM‐CSF and Flt3L to generate CD103^+^ dendritic cells (**Figure** **6**A).^[^ ^20^ ^]^ Bone marrow‐derived dendritic cells (BMDCs) from *Irf8*‐OE mice exhibited a more mature phenotype, characterized by extensive protrusions and larger cell size (Figure S6A, Supporting Information). Enrichment analysis of transcriptomic sequencing data from activated BMDCs revealed significant differences between the *Irf8*‐OE and control mice (*Irf8* ^CAG‐LSL^). KEGG and Reactome analyses of the differentially expressed genes (DEGs) highlighted inflammatory processes and alterations in ECM components in the *Irf8*‐OE samples (Figure S6B,C, Supporting Information). GO analysis uncovered several biological processes and molecular functions—including T cell activation and chemotaxis—that were upregulated in the BMDCs from *Irf8*‐OE mice, indicating the role of IRF8 in the interaction between DCs and T cells (Figure 6B). Upregulated DEGs with potential interactions with IRF8, as identified by the STRING database (e.g., *Cd40* and *Fcgr1*), were included in the protein–protein interaction network analysis (Figure S6D, Supporting Information), suggesting that IRF8 may regulate the expression of key surface markers involved in antigen presentation by BMDCs.^[^ ^21^ ^]^ The biological processes enriched with the IRF8‐related DEGs were predominantly associated with type I interferon signaling pathways, which play substantial roles in the antigen‐presentation functions of dendritic cells (Figure 6C).^[^ ^22^ ^]^ To evaluate the effect of IRF8 on the antigen‐presentation function of cDC1s at the protein level, we isolated BMDCs treated with either control or *Irf8* siRNA and stimulated them with TNF‐α (Figure 6D,E). Our results revealed that CD103^+^ cDC1s with *Irf8* silencing exhibited reduced expression of CD64 (encoded by the *Fcgr1* gene), a molecule previously shown to be crucial for antigen cross‐presentation to CD8^+^ T cells during early‐stage immune responses.^[^ ^23^ ^]^

### 2.7 IRF8 Expression Properties in Human Affects AAA Development

**Figure 7:**
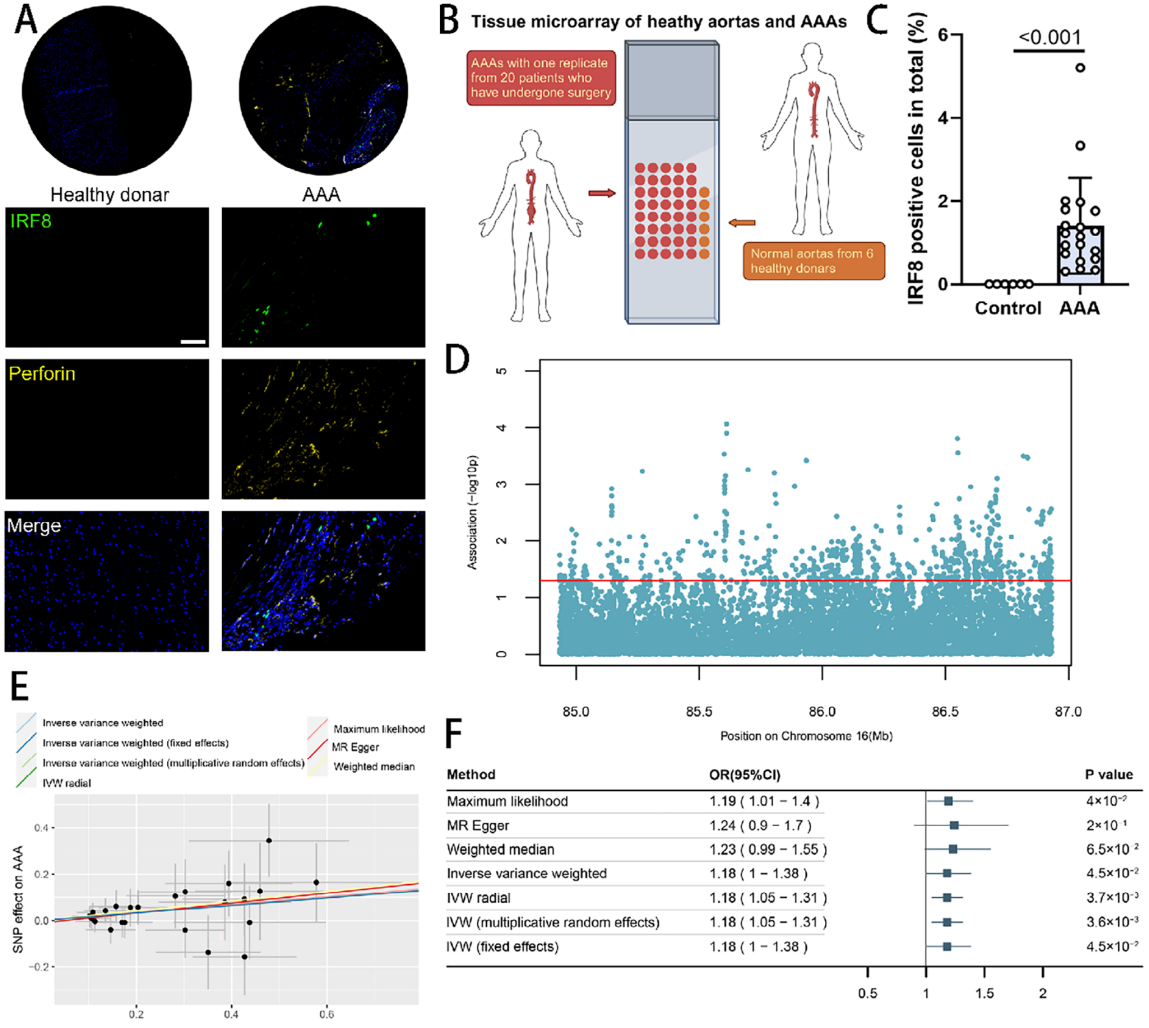
IRF8 expression properties in humans affect AAA development. A) Representative images of multiplex immunohistochemistry imaging for the IRF8 marker (green) and perforin (yellow) marker in the tissue microarray made by 26 human aortic samples (20 AAA samples and 6 healthy aortas, scale bar 50 µm), suggesting a higher expression level of IRF8 and extended perforin^+^ region in the human AAA samples. B) Graphic illustration of the preparation of tissue microarray. AAA samples were acquired from 20 patients with one replicate, and healthy controls were derived from 6 healthy donors. C) Quantification of the IRF8 expression level in the tissue microarray made by 26 human aortic samples (20 AAA samples and 6 healthy aortas, scale bar 50 µm) (*n* = 20 in the AAA group, and *n* = 6 in healthy aortas, unpaired two‐tailed Student's *t*‐test). D) Association with abdominal aortic aneurysm of variants within 50 kb of the *IRF8* transcript (Manhattan plot). E) Scatter plot of the Mendelian randomization analysis showing the causal estimates of the variants of *IRF8* on AAA development. F) Forest plot of MR results between *IRF8* eQTLs and AAA utilizing maximum likelihood, MR Egger, weighted median and Inverse variance weighted (*n* = 24). To investigate the causal relationship between genetic IRF8 expression and abdominal aortic aneurysm, common variants associated with IRF8 expression (minor allele frequency ≥ 0.01) from the Genotype‐Tissue Expression (GTEx) Project Version 7 were selected. IRF8, interferon regulatory factor 8; AAA, abdominal aortic aneurysm; MR, Mendelian randomization; eQTL, expression quantitative trait locus.

To explore the influence of IRF8 on human AAA expansion, we designed a tissue microarray comprising AAA tissue from 20 patients undergoing surgery and 6 normal aortas from healthy donors. Multiplexed immunohistochemistry marking IRF8 and perforin in aortic samples exhibited widespread presence of perforin in the AAA samples (Figure **7**A,B). Additionally, compared with normal aortas, AAA sections exhibited significantly higher expression levels of IRF8 (Figure 7C), suggesting the profound impact of IRF8 on human AAA occurrence. To further clarify the relationship between IRF8 expression quantitative trait loci (eQTL) and human AAA incidence, we analyzed genome‐wide association study (GWAS) data from the pan‐ancestry genetic analysis of the UK Biobank. AAA was defined as Phenocode 442.11, including 1306 cases and 408 565 controls of both sexes. Multiple Mendelian randomization (MR) methods were employed to assess causal effects, including inverse variance weighted, MR‐Egger, weighted median, and maximum likelihood. Sensitivity analyses assessed pleiotropy using the MR‐Egger intercept *P* value and quantified heterogeneity with Cochran's Q statistic (Figure S7A,B, Supporting Information). Variants throughout the *IRF8* locus demonstrated significant associations with AAAs (Figure 7D; and Figure S7C, Supporting Information). MR results showed that IRF8 has a causal effect on the development of AAA (Figure 7E,F). Additionally, the association between plasma Granzyme B levels and AAA (IEU Open GWAS program, dataset prot‐a‐1297) suggests that patients with AAA exhibit elevated expression of cytotoxic proteins (see Table S1, Supporting Information). Collectively, these findings show consistency between murine models and human data in AAA pathogenesis.

## 3 Discussion

In this study, we have elucidated the pivotal role of IRF8 in the pathogenesis of AAA. Our findings demonstrate that IRF8 expression is significantly upregulated in both human and murine AAA samples, and that IRF8 activation promotes the differentiation of cDC1s, leading to the recruitment and activation of CD8^+^ T cells. This cascade contributes to the destruction of the aortic wall and the development of AAA.

The expansion of cDC1s and the subsequent activation of CD8^+^ T cells suggest a specific immunological pathway in AAA pathogenesis. cDC1s are known for their ability to cross‐present antigens to CD8^+^ T cells.^[^ ^24^ ^]^ Previous studies have explored the roles of cDC1s and CD8^+^ T lymphocytes in cardiovascular diseases. For instance, Krishna et al. demonstrated that the depletion of CD11c^+^ cells inhibited experimental AAA development and growth, which was associated with the downregulation of circulating effector T cells and attenuation of matrix degradation.^[^ ^10^ ^]^ Similarly, Clément et al. found that restricted deletion of IRF8 in CD11c^+^ cells—resulting in the ablation of lymphoid CD8α^+^ cDC1s and aortic CD11b^−^CD103^+^ cDC1s—led to profound inhibition of T cell activation and differentiation in response to a high‐fat diet, reducing atherosclerosis.^[^ ^15^ ^]^ In human AAAs, it was also reported that the CD8^+^ T cell population is upregulated, which exhibited a positive correlation associated with AAA size and severity.^[^ ^25^ ^]^ IFN‐γ production by CD8+ T cells was found to promote the dilation of AAA induced with elastase perfusion.^[^ ^26^ ^]^ Additionally, the deletion of CD8^+^ T cells was shown to have vascular protective effects in a mouse CaCl_2_‐induced AAA model due to the attenuation of IFN‐γ‐dependent inflammatory actions, oxidative stress production, and proteolysis.^[^ ^27^ ^]^ Single‐cell RNA sequencing of the angiotensin II infusion model uncovered a significant increase in cDC1 infiltration across varying stages of AAA samples.^[^ ^28^ ^]^ Our recent single‐cell transcriptomic study of murine AAAs induced with periadventitial elastase also revealed that the proportion of cDC1s differed significantly between control and aneurysm groups, accompanied by increased proliferation and differentiation of CD8^+^ T cells into effector cells.^[^ ^29^ ^]^ This suggests a critical role for cDC1s and CD8^+^ T cells in AAA development.

Building on these findings, our current study demonstrates that IRF8 and BATF3 influence the progression of AAA by regulating the differentiation and infiltration of cDC1s. Notably, IRF8 appears to have a more significant role than BATF3, as evidenced by its higher differential expression in human aneurysm tissue microarrays compared to healthy tissue. The enhanced differentiation of cDC1s leads to increased activation of CD8⁺ T cells, which contribute to aortic wall degradation in AAA primarily through apoptosis‐mediated cell death. Furthermore, targeting cDC1s via CLEC9A neutralizing antibodies effectively inhibited the expansion of AAA in mice, reinforcing the notion that CD8⁺ T cells are activated by cDC1s to promote AAA progression. Fc gamma receptors are expressed in conventional dendritic cells and play a critical role in enhancing antigen uptake and presentation.^[^ ^30^ ^]^ In our in vitro studies, we demonstrated that IRF8 promotes the expression of FcγRI (CD64) in cDC1s, a receptor known to facilitate cross‐presentation to CD8^+^ T cells, particularly during the early stages of immune responses.^[^ ^23^ ^]^ By elucidating the IRF8‐cDC1‐CD8⁺ T cell axis and highlighting CLEC9A as a potential therapeutic target, we provide novel insights into the immunological mechanisms underlying AAA pathogenesis.

The pathogenesis of AAA is closely linked to the degradation of the ECM, a process critically mediated by (MMPs, particularly MMP2 and MMP9.^[^ ^31^ ^]^ MMP2, primarily secreted by fibroblasts and SMCs, is responsible for the degradation of collagen and elastin, while MMP9, mainly produced by macrophages, drives inflammatory ECM remodeling.^[^ ^32^ ^]^ Despite their essential roles in ECM breakdown, clinical trials targeting these MMPs have consistently failed to yield effective therapies for AAA,^[^ ^33^ ^]^ suggesting that inhibition of MMP activity alone does not sufficiently address the complex pathology of AAA. Our study demonstrates that cDC1s and CD8⁺ T cells induce apoptosis in SMCs, which in turn contributes to the degradation of the ECM with disrupted elastin and increased collagen deposition, accompanied by increased gelatinase activity and cathepsin expression. This interplay between direct proteolytic activity and immune‐mediated cell death underscores the multifaceted nature of ECM remodeling in AAA and highlights the need for therapeutic strategies that target both proteolytic enzymes and immune cell‐driven pathways.

The current approach to managing asymptomatic AAA treatment usually includes regular imaging to monitor aneurysm size and growth. Both patients and clinicians need effective drug therapies to slow AAA growth and minimize the risks of repair and rupture.^[^ ^34^ ^]^ Recent studies have highlighted the potential of nanotherapy targeting immune cells to inhibit AAA expansion, paving the way for translating basic research into clinical applications.^[^ ^35^ ^]^ Research has increasingly focused on the roles of rare immune cells within the AAA microenvironment. For example, eosinophils have been shown to play a protective role in limiting AAA progression by modulating macrophage and monocyte polarization.^[^ ^36^ ^]^ Similarly, group 2 innate lymphoid cells contribute to AAA prevention in mice by promoting eosinophil activation through IL‐5 signaling.^[^ ^37^ ^]^ These findings underscore the significance of immune cell interactions in AAA pathogenesis. Our own human studies reinforce the translational relevance of these immune‐mediated mechanisms. We observed elevated levels of IRF8 and perforin in AAA patient samples, mirroring murine data and suggesting that the IRF8‐cDC1‐CD8^+^ T cell axis is similarly active in human disease. Furthermore, Mendelian randomization analyses using GWAS data from the UK Biobank further suggest a causal relationship between IRF8 expression and AAA development, highlighting potential therapeutic targets within the IRF8‐cDC1‐CD8^+^ T cell pathway.IRF8‐cDC1‐CD8. These insights open new avenues for developing prevention and treatment strategies for asymptomatic AAAs.

Future research should aim to elucidate the upstream regulators of IRF8 in the aortic wall and identify potential triggers, such as mechanical stress or cytokine signals that may influence its expression. Investigating the interplay between IRF8 and other immune pathways could reveal additional therapeutic targets. Clinical studies involving larger patient cohorts are necessary to validate IRF8 as a biomarker for AAA progression and to assess the efficacy of IRF8‐targeted therapies. Exploring combination treatments that address multiple aspects of the immune response may enhance therapeutic outcomes.

Our study demonstrates that IRF8 plays a crucial role in AAA development by promoting cDC1 differentiation and activating CD8^+^ T cells, leading to aortic wall destruction. These insights advance our understanding of the immunopathology of AAA and highlight potential avenues for therapeutic intervention targeting the IRF8‐mediated immune response.

## 4 Experimental Section

### Human AAA Study

Human AAA specimens were obtained from patients undergoing open AAA repair surgery, while normal aortic samples were sourced from organ transplantation donors. All procedures adhered to ethical guidelines for the treatment of human specimens and received approval from the Human Research Ethics Committee of the Second Affiliated Hospital, Zhejiang University School of Medicine. Written informed consent was obtained from all participants, and the studies conformed to the principles outlined in the Declaration of Helsinki. The study utilizing the UK Biobank (UKB) was approved by the North West Multicenter Research Ethics Committee (approval number: 11/NW/0382). All participants provided written informed consent prior to inclusion. Genotypic and clinical data were sourced from the UKB datasets in accordance with application number 105 945.

### Animals

All mice were male and on the C57BL/6 background. *Batf3* ^−/−^, *Clec9a* ^−/−^, *Irf8* ^flox/flox^, and *Itgax* ^Cre^ mice were generated by GemPharmatech Co., Ltd (Nanjing, China). *Irf8* ^flox/flox^ mice were crossed with *Itgax* ^Cre^ mice to generate DC‐specific *Irf8*‐knockout mice (*Irf8* ^flox/flox^ *Itgax* ^Cre^, abbreviated to *Irf8* ^ΔDC^ in the following paragraphs). Using CRISPR/Cas9 technology, *Irf8* (H11‐CAG‐LSL‐*Irf8*‐Flag‐polyA) was knocked into the WT mice, to generate *Irf8* ^CAG‐LSL^ mice (GemPharmatech Co., Ltd), which were then crossed with *Itgax* ^Cre^ mice to generate DC‐specific *Irf8*‐overexpressing mice (*Irf8* ^CAG‐LSL^ *Itgax* ^Cre^, abbreviated to *Irf8*‐OE in the following paragraphs). Detailed information on gene‐modified mice can be found in the Supporting Information_ Gene‐modified mice.

To minimize selection bias, animals were randomly assigned to experimental groups using a computer‐generated randomization sequence with GraphPad Prism 9.0 software. The allocation sequence was generated by an investigator not directly involved in data collection or analysis. Cage location or housing was randomized to ensure equal environmental exposure across groups. Allocation concealment was maintained until the completion of the experiment to avoid potential biases during group assignment. The baseline heart rate and systolic blood pressure were measured and listed in Table S2 (Supporting Information), and representative reports of complete blood count were exhibited in Table S3 (Supporting Information), to avoid bias caused by differences in fundamental conditions among distinct genotypes of mice. To reduce the risk of bias, all outcome assessments were performed in a blinded manner. Blinding was maintained by labeling groups with randomly assigned codes, which were only revealed after data analysis was completed. Additionally, during data collection, the observer was not involved in the randomization process or allocation of animals to ensure unbiased assessment. Specific criteria for inclusion and exclusion were established prior to the start of the study. Animals were included if they were of the specified species, strain, age, and sex and displayed no signs of pre‐existing illness or injury at the start of the study. Exclusion criteria included animals with unexpected health conditions, failure to reach the predefined weight range, or injuries unrelated to the experimental procedures. All inclusion and exclusion criteria were consistently applied across all experimental groups.

Throughout the animal experiments, mice were maintained under standard housing conditions, including a 12‐h light/dark cycle, with ad libitum access to food and water. All animal care and experimental procedures were conducted in accordance with the guidelines set by the Institutional Animal Care and Use Committee at Zhejiang University College of Medicine, ensuring ethical and humane treatment of the animals.

### Murine Model of Elastase‐Induced AAA

AAA was induced by periadventitial application of elastase, which was reported to mimic human AAA pathology,^[^ ^38^ ^]^ as previously described.^[^ ^39^ ^]^ Briefly, 8‐week‐old male mice were anesthetized via intraperitoneal injection of a ketamine (50 mg kg^−1^) and pentobarbital sodium (50 mg kg^−1^) mixture. A midline abdominal incision was made to expose and isolate the infrarenal segment of the abdominal aorta. This segment was wrapped with sterile cotton soaked in 10 µL of porcine pancreatic elastase (E1250, Sigma‐Aldrich) for the experimental group or heat‐inactivated elastase for the sham group, and left in place for 10 min. After elastase application, the cotton was removed, and the abdominal cavity was flushed twice with saline. The incision was then carefully sutured closed. AAA samples were collected 14 days postprocedure. Aortic dilation was quantified as the percentage increase in aortic diameter compared to the initial measurement, calculated using the formula: [(Final measurement − Initial measurement)/Initial measurement] × 100%, as previously reported.^[^ ^40^ ^]^ The absolute measurements of the aortic diameters are listed in Table S4 (Supporting Information).

### Angiotensin II‐Induced Murine AAA Model

An Ang II mini pump model was established following previously reported methods.^[^ ^41^ ^]^ Briefly, Alzet osmotic minipumps (Model 1004; ALZA Scientific Products, Cupertino, CA) were implanted in 8‐week‐old male *Apoe* ^−/−^ mice. The pumps, loaded with either saline or Ang II solutions (Sigma Chemical Co.), were programmed to deliver Ang II at 1000 ng min^−1^ kg^−1^ subcutaneously for 28 days. For pump implantation, mice were anesthetized with a ketamine/xylazine mixture. A small incision was made in the posterior neck region to insert the pump into the subcutaneous space, which was then sealed with surgical glue. The incision sites healed quickly, eliminating the need for further medication.

### In Vivo Antibody Administration

Neutralizing antibody administration was performed as previously described.^[^ ^42^ ^]^ Mice received intraperitoneal injections of 400 µg of anti‐CLEC9A antibody (clone 7H11, BioXCell) or the corresponding isotype controls every other day, starting on the day of surgery.

### Histological Studies

Human and murine aortic tissues were paraffin‐embedded and sectioned into 5 µm slices, while OCT‐embedded frozen samples were cut into 7 µm slices. Van Gieson staining was performed using the Verhoeff–Van Gieson kit (Jisskang Biotechnology Co. Ltd, China) to assess elastin degradation, following the manufacturer's protocol. Images were captured using a Leica 2500 microscope. The degree of elastin degradation was evaluated using a previously established scoring system: I (no or mild elastin degradation), II (moderate elastin degradation), III (moderate to severe elastin degradation), and IV (severe elastin degradation).^[^ ^43^ ^]^

### Masson Staining

Collagen deposition was assessed using Masson's Trichrome Staining Kit (Beyotime, China). Tissue sections were stained with hematoxylin for 5 min, washed, differentiated for 30 s, and rinsed for 10 min. Lichun Red‐Acid Fuchsin staining was applied for 10 min, followed by a brief rinse and 2‐min differentiation. Light Green staining was performed for 1 min, followed by a rinse and 1‐min differentiation. Sections were dehydrated in graded ethanol for 10 s, cleared in xylene (3 × 1–2 min), and mounted with neutral balsam for microscopic examination.

### Immunofluorescence Staining

Antigen retrieval was performed on paraffin‐embedded AAA sections prior to staining. Sections were permeabilized for 10 min with 0.1% Triton X‐100 after fixation with 0.4% paraformaldehyde. After rinsing with phosphate‐buffered saline (PBS), samples were incubated for 1 h in a blocking solution containing 10% serum from the species of the secondary antibody (e.g., goat or donkey). Primary antibodies were applied overnight at 4 °C. Following PBS washes, sections were incubated with secondary antibodies or the TUNEL kit (C1090, Beyotime, China) for 1 h at room temperature. Nuclei were counterstained and mounted using Antifade Mounting Medium with DAPI (P0131, Beyotime, China). Imaging was performed using a Leica DM6B microscope for epifluorescence, brightfield, and polarized light microscopy. Image analysis and processing were conducted using the provided software, Adobe Photoshop 2023, and ImageJ (https://ij.imjoy.io/; National Institutes of Health).

### Flow Cytometry

Aortic tissues were collected and minced into small pieces. Single‐cell suspensions were prepared using Aorta Dissociation Enzyme Solution (ADES), following established protocols.^[^ ^44^ ^]^ The suspensions were incubated with an Fc blocker for 5 min at room temperature, and then stained with specific antibodies for 30 min. After washing, fluorescence was measured using a BD FACS Canto II Flow Cytometer (BD Biosciences). Data were analyzed using FlowJo v10 software (Tree Star). Leukocytes were identified in SSC‐A/CD45 dot plots as CD45‐positive cells.

### Western Blot Analysis

Cells or aortic tissues were harvested and lysed using RIPA lysis buffer (Beyotime) supplemented with protease inhibitors. Protein concentrations were determined using the BCA assay (Invitrogen). Equal amounts of protein were loaded onto SDS‐PAGE gels for electrophoresis, followed by transfer onto PVDF membranes. Membranes were incubated with primary antibodies at 4 °C overnight, then with secondary antibodies for 1 h at room temperature. Protein bands were visualized using a western blot detection system, including the ChemiDoc MP (Bio‐Rad) and Amersham ImageQuant 800 (Cytiva). Data were normalized against internal controls to ensure accuracy and consistency.

### In Situ Zymography

Gelatinase activity was quantified in situ by visualizing fluorescence resulting from the proteolytic cleavage of intramolecularly quenched fluorophore‐labeled DQ‐gelatin, utilizing the EnzChek Gelatinase/Collagenase Assay Kit (Molecular Probes). In brief, 10‐µm frozen tissue sections were equilibrated to room temperature to ensure optimal interaction with the substrate. Each section was then overlaid with 20 µL of a solution containing 100 µg mL^−1^ DQ‐gelatin in 1X reaction buffer, which was supplemented with protease inhibitors to avoid the impact of gelatinase activity. The sections were covered with a glass coverslip, allowed to gel for 5 min at 4 °C, and subsequently incubated for 4 h at 37 °C protected from light. To assess the specificity of gelatinase activity, some sections were incubated in the presence of the metalloproteinase inhibitor EDTA at a final concentration of 10 mmol L^−1^ as negative controls. Fluorescent products resulting from the enzymatic cleavage of DQ‐gelatin exhibit an absorption maximum at ≈495 nm and an emission maximum at ≈515 nm, which were detected by a fluorescence microscope to assess gelatinase activity.

### Tissue Microarray

Abdominal aortic tissues were obtained from patients who underwent surgery at our department. A total of 26 patients from the Second Affiliated Hospital of Zhejiang University were recruited for the study, all of whom provided informed consent. Tissue microarrays (TMAs) were constructed using a manual tissue array, comprising 20 AAAs and 6 healthy aortas from donors, all embedded in paraffin blocks. For the TMA, two replicate cores were taken from each aneurysmal tissue and one core from each healthy aorta. Sections of 4 µm thickness were sliced and stained with antibodies. To ensure consistency, three randomly selected fields per condition were imaged using identical microscope settings. Images were scanned and analyzed using Caseviewer software.

### Multiplexed Immunohistochemistry

Multiplexed immunohistochemistry was performed on 4‐µm‐thick formalin‐fixed, paraffin‐embedded tissue sections using sequential primary antibody staining and the TSA 7‐color kit (abs50015‐100T, Absinbio), followed by nuclear staining with DAPI. Deparaffinized slides were incubated with anti‐IRF8 antibody (#306 552, Abcam) for 30 min, followed by a 10‐min application of antirabbit/mouse horseradish peroxidase‐conjugated secondary antibody (#A10011‐60, Absinbio) and developed for 10 min using the TSA 520 protocol. Slides were rinsed with TBST buffer, immersed in preheated citrate solution at 90 °C, and heat‐treated in a microwave at 20% power for 15 min before cooling to room temperature. Between each step, slides were washed with Tris buffer. The procedure was repeated for subsequent antibodies and fluorescent dyes, specifically antiperforin (PA5‐87351, Invitrogen) with TSA 570. The microarray was then stained with DAPI (D1306, Thermo Fisher), rinsed with distilled water, and cover‐slipped manually. After air drying, slides were imaged using an Aperio Versa 8 system (Leica) and analyzed with Indica Halo software.

### Genome‐Wide Association Study Data

Summary statistics from the pan‐ancestry genetic analysis of the UK Biobank were included to address potential biases in multiancestry studies. AAA was defined using Phenocode 442.11, comprising 1306 cases, and 408565 controls of both sexes. Detailed information is available at UK Biobank Resource.

### Data Availability

The datasets and computer code supporting the findings are available from the corresponding author upon reasonable request. The RNA sequencing datasets have been deposited in the Gene Expression Omnibus under accession number GSE278890. Other datasets implicated in this study were acquired from GSE183464,^[^ ^45^ ^]^ GSE232911,^[^ ^16^ ^]^ and GSE237067.^[^ ^29^ ^]^

### Statistical Analysis

The normality of data distribution was assessed using the Shapiro–Wilk test in GraphPad Prism 9.0, and homogeneity of variances was tested using Levene's test. Values are presented as means ± standard error of the mean. For comparisons between two groups with normal distribution and equal variances, an unpaired two‐tailed Student's *t*‐test was used; otherwise, the Mann–Whitney U test was applied. For comparisons involving three groups, a one‐way analysis of variance (ANOVA) was conducted if normality and homogeneity of variances were satisfied, followed by Tukey's Honest Significant Difference post‐hoc test for pairwise comparisons. When these assumptions were violated, the Kruskal–Wallis test was performed as a nonparametric alternative. *P*‐values less than 0.05 were considered statistically significant.

### Ethical Statement

All animal care and experimental procedures were following the Ethics Committee rules of the Institute following the current European legislation (Council Directive 86/609/EEC; Law 5/1995/GC; Order 214/1997/GC; Law 1201/2005/SG) and the guidelines of the Institutional Animal Care and Use Committee at Second Affiliated Hospital, Zhejiang University College of Medicine.

## Conflict of Interest

The authors declare no conflict of interest.

## Supporting information



Supporting Information

## Data Availability

The data that support the findings of this study are openly available in The regulatory effect of IRF8 on the gene expression profiles of bone marrow derived dendritic cells at http://www.ncbi.nlm.nih.gov/geo/query/acc.cgi?acc=GSE278890, reference number 278890.
