# Supplementary material for: IRF8 Drives Conventional Type 1 Dendritic Cell Differentiation and CD8+ T Cell Activation to Aggravate Abdominal Aortic Aneurysm Development
Source: Adv Sci (Weinh). 2025 Apr 4;12(22):2416238. doi: 10.1002/advs.202416238 (PMC12165085; doi:10.1002/advs.202416238)
Supplement: Supplementary file 1 — Supporting Information [file ADVS-12-2416238-s001.docx]

**Supplemental Methods**

**Animals**

All mice were male and on the C57BL/6 background. *Batf3*^-/-^, *Clec9a*^-/-^, *Irf8*^flox/flox^, and *Itgax*^Cre^ mice were generated by GemPharmatech Co., Ltd (Nanjing, China). *Irf8*^flox/flox^ mice were crossed with *Itgax*^Cre^ mice to generate DC-specific *Irf8*-knockout mice (*Irf8*^flox/flox^*Itgax*^Cre^, *Irf8*^ΔDC^). Using CRISPR/Cas9 technology, *Irf8* (H11-CAG-LSL-*Irf8*-Flag-polyA) was knocked into the WT mice, to generate *Irf8*^CAG-LSL^ mice (GemPharmatech Co., Ltd), which were then crossed with *Itgax*^Cre^ mice to generate DC-specific *Irf8*-overexpressing mice (*Irf8*^CAG-LSL^*Itgax*^Cre^, *Irf8*-OE). The complete blood count was analyzed with Mindray Veterinary automated hematology analyzer model (bc-2800vet). Arterial systolic blood pressure and heart rate were measured using VisualSonics Vevo 2100 Imaging System.

**Cell Isolation and Cultivation**

The protocol for acquiring bone marrow-derived dendritic cells (BMDCs) was detailed in a previous study (1). Briefly, 8-week-old male mice were euthanized by cervical dislocation, and their legs were dissected. After removing the surrounding muscle tissue, bone marrow was extracted aseptically from the tibia and femur. This was done by cutting the joints with a scalpel and flushing the marrow out with a 25-gauge needle attached to a syringe filled with 10 ml of PBS. The marrow clumps were then gently dissociated using a needle-free syringe through a 70 μm cell strainer. The cell suspension was centrifuged at 1000 g for 5 minutes at room temperature to pellet the cells. The collected bone marrow cells (15x10^6^) were resuspended in 10 ml of RPMI 1640 growth medium, supplemented with 10% fetal bovine serum (FBS), 2 mM L-glutamine, 100 units/ml penicillin, 0.1 mg/ml streptomycin, 200 ng/ml recombinant murine FLT3L (14-8001, eBioscience), and 5 ng/ml granulocyte-macrophage colony-stimulating factor (GM-CSF, 315-03, Peprotech). On days 5 to 6, an additional 5 ml of complete medium was added to reduce apoptosis. Non-adherent cells were harvested on day 9, counted, and replated at a density of 3x10^6^ cells in 10 ml of complete medium with FLT3L and GMCSF, as on day 0. Non-adherent iCD103-DCs were collected on days 15-16. The cells could be stimulated with 20 ng/ml of TNF-alpha.

**RNA Sequencing and Bioinformatic Analysis**

RNA integrity was evaluated with the Agilent Bioanalyzer 2100 system. mRNA was extracted from total RNA using magnetic beads and then fragmented using divalent cations. First-strand cDNA synthesis involved a random hexamer primer and M-MuLV Reverse Transcriptase, followed by second-strand synthesis using DNA Polymerase I and RNase H. After processing ends and adding adapters, cDNA fragments of 370-420 bp were selected and PCR-amplified. The PCR products' quality was verified using the Bioanalyzer system. The index-coded samples underwent clustering on a cBot system and were sequenced on an Illumina Novaseq platform, yielding 150 bp paired-end reads. For Bone Marrow-Derived Dendritic Cells (BMDCs), genes were considered to show significant differential expression if they had a False Discovery Rate (FDR) corrected P-value of less than 0.05 and an absolute log2 fold change greater than 1, indicating a 2-fold change.

**ImmGen analysis of human AAA samples**

In accordance with the literature "Tunica-Specific Transcriptome of Abdominal Aortic Aneurysm and the Effect of Intraluminal Thrombus, Smoking, and Diameter Growth Rate," differentially expressed genes common to both the medial layer and adventitial layer were identified (|FC| ≥ 1 and adjusted p ≤ 0.05). These genes were then intersected with the human transcription factor gene set from the AnimalTFDB database (https://guolab.wchscu.cn/AnimalTFDB4/#/). A total of nine transcription factors were found to be differentially expressed in both the medial and adventitial layers. The relative expression levels of these transcription factors across various immune cells (ImmGen Microarray V1) were subsequently analyzed using the Databrowser tool in the ImmuGen database (<https://rstats.immgen.org/MyGeneSet_New/index.html>).

**Mendelian randomization analysis**

Mendelian randomization (MR) analyses were conducted using the TwoSampleMR R package (version 0.6.4), with IRF8 expression level as the exposure and abdominal aortic aneurysm as the outcomes.

Instrumental variables (IVs) were selected based on genome-wide significance to minimize confounding from variants directly associated with the outcome but not through the exposure. To avoid weak instruments, IVs with an F statistic less than 10 were excluded.

Multiple MR methods were employed to assess causal effects, including inverse variance weighted (IVW), MR-Egger, weighted-median, maximum likelihood. The primary analysis used the IVW method with a random-effects model. MR-Egger analysis was used to provide robust estimates in the presence of horizontal pleiotropy. The weighted median method accounted for heterogeneity and outliers. Sensitivity analyses assessed pleiotropy using the MR-Egger intercept P value and quantified heterogeneity with Cochran’s Q statistic.

To investigate the causal relationship between genetic IRF8 expression and abdominal aortic aneurysm, common variants associated with IRF8 expression (minor allele frequency ≥ 0.01) from the Genotype-Tissue Expression (GTEx) Project Version 7 were selected. To investigate the causal relationship between genetic Granzyme B plasma levels and abdominal aortic aneurysm, common variants associated with their expression was extracted from IEU Open GWAS program, dataset prot-a-1297. After excluding variants in high linkage disequilibrium, Mendelian randomization was performed, taking linkage disequilibrium between variants into account.

**Reference**

1. Mayer CT, Ghorbani P, Nandan A, Dudek M, Arnold-Schrauf C, Hesse C, et al. Selective and efficient generation of functional Batf3-dependent CD103+ dendritic cells from mouse bone marrow. Blood. 2014;124(20):3081-91.

**Supplementary Figures**

**
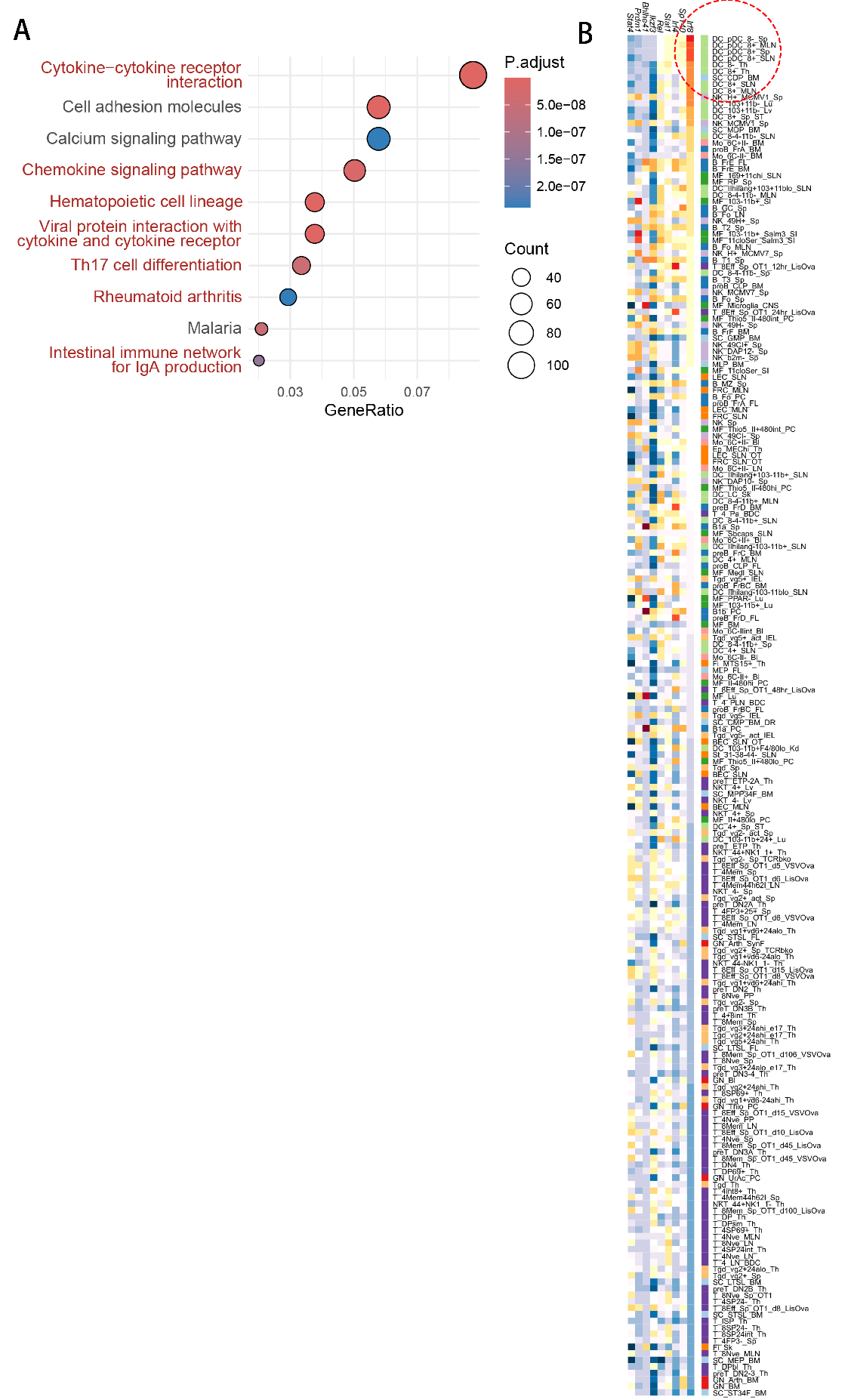
**

**Figure S1** (A) Dot plot of KEGG enrichment analyses for the transcriptional level of upregulated genes of human AAA tissue compared with healthy aortas (male, GSE183464 dataset). (B) Heatmap exhibiting the distribution of remarkable DEGs from the human AAA tissue compared with healthy aortas using ImmGen.


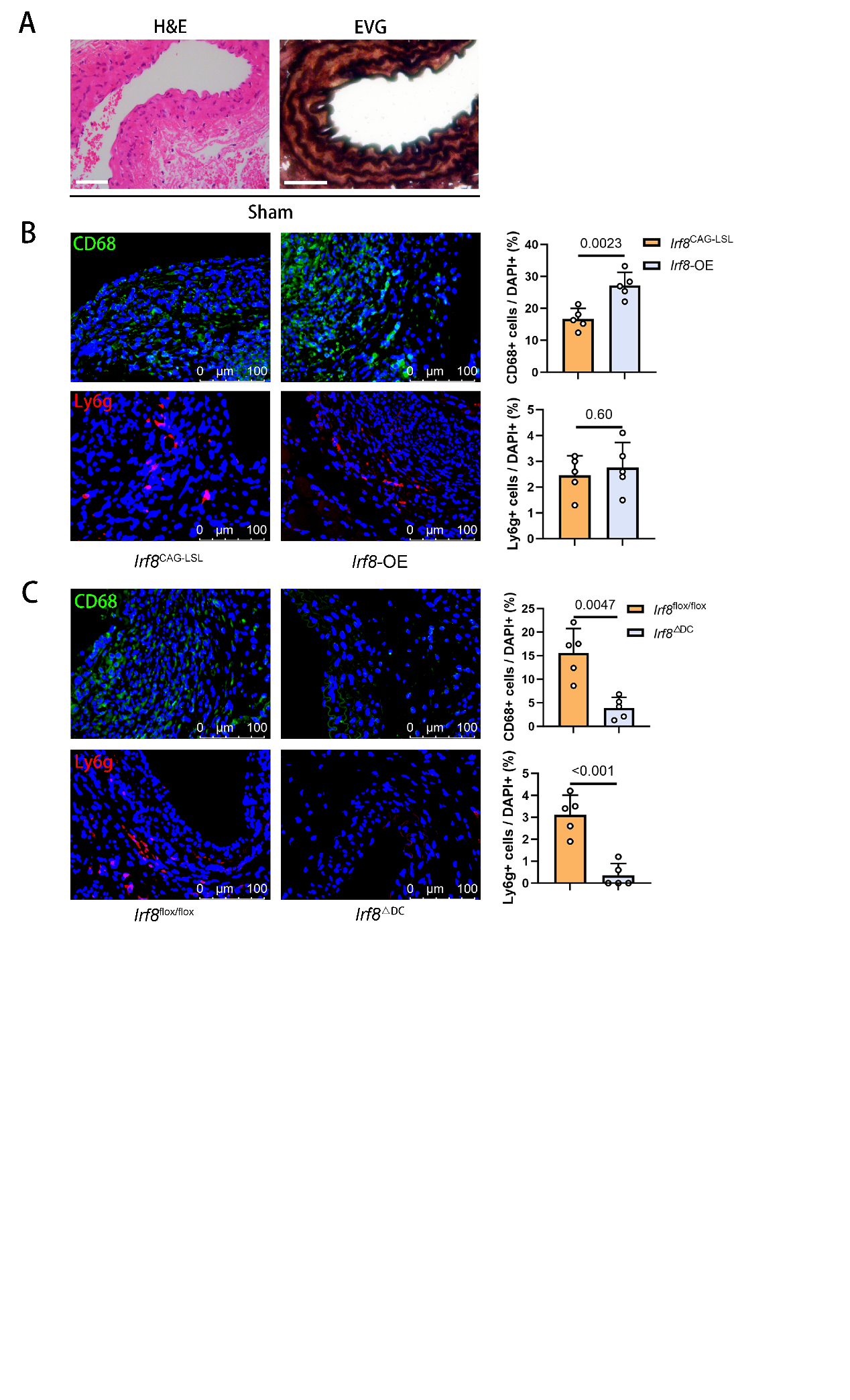


**Figure S2** (A) Representative images of histology of abdominal aortas of the sham group. Scale bar: 50μm for H&E staining (40x) and elastin Van Gieson (63x). (B) Representative immunofluorescence images and quantitative analysis of aortic sections from the control and *Irf8*-OE group 14 days after peri-adventitial elastase application with staining for CD68 (green), Ly6g (red) and nuclei (blue) (n = 5 of each group). Scale bar, 100 μm. (C) Representative immunofluorescence images and quantitative analysis of aortic sections from the control and *Irf8*^ΔDC^ group 14 days after peri-adventitial elastase application with staining for CD68 (green), Ly6g (red) and nuclei (blue) (n = 5 of each group). Scale bar, 100 μm.

**
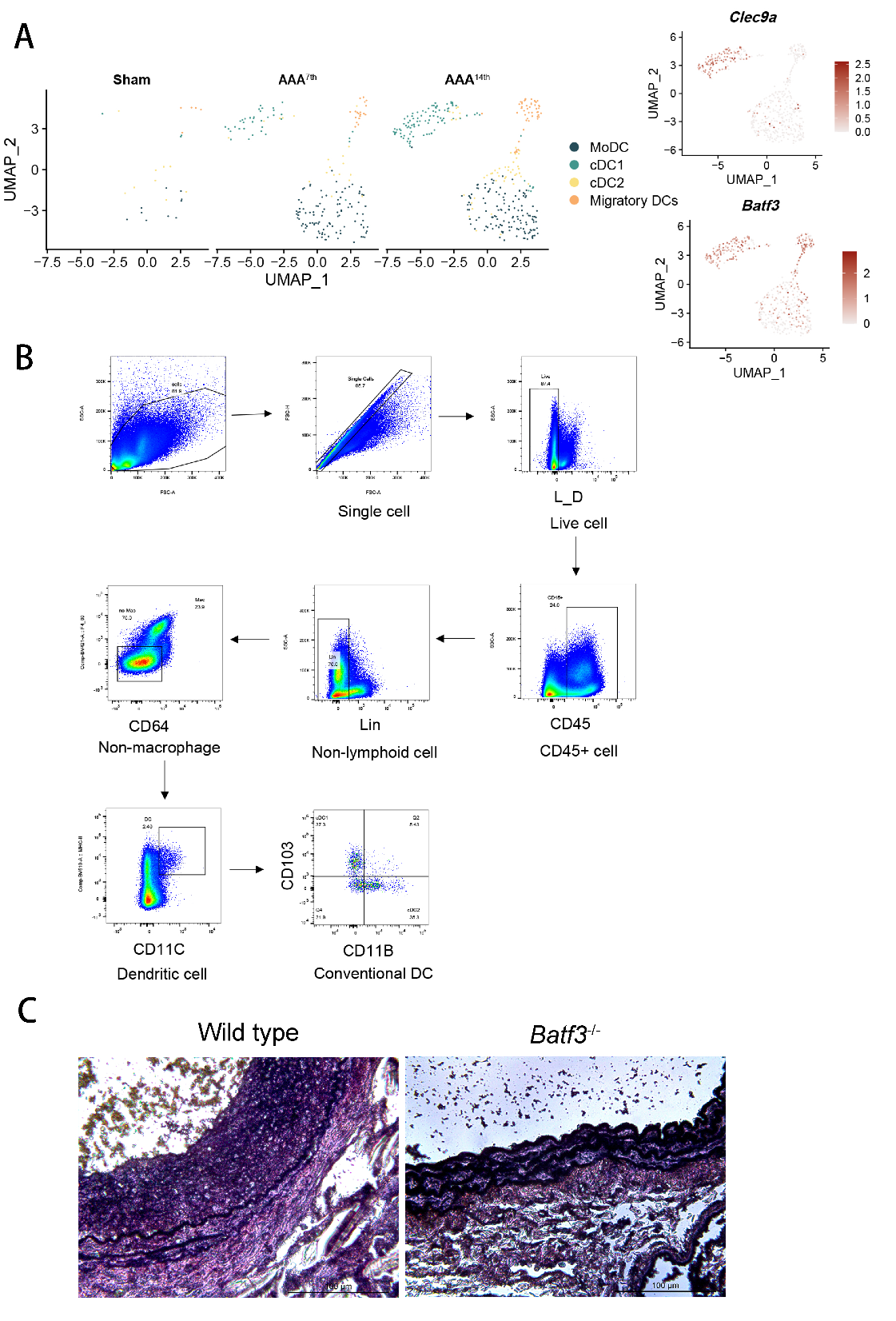
**

**Figure S3** (A) UMAP visualization of the DC subpopulations of murine AAA samples at day 0, day 7 and day 14 post-surgery and gene expression profiles of *Batf3* and *Clec9a* among DC subtypes. (B) Gating information for flow cytometry of dendritic cells in murine AAA samples. (C) Representative images of elastin Van Gieson staining of abdominal aortas of the control and *Batf3* knock-out groups 14 days after application of peri-adventitial elastase. Scale bar: 100μm.

**
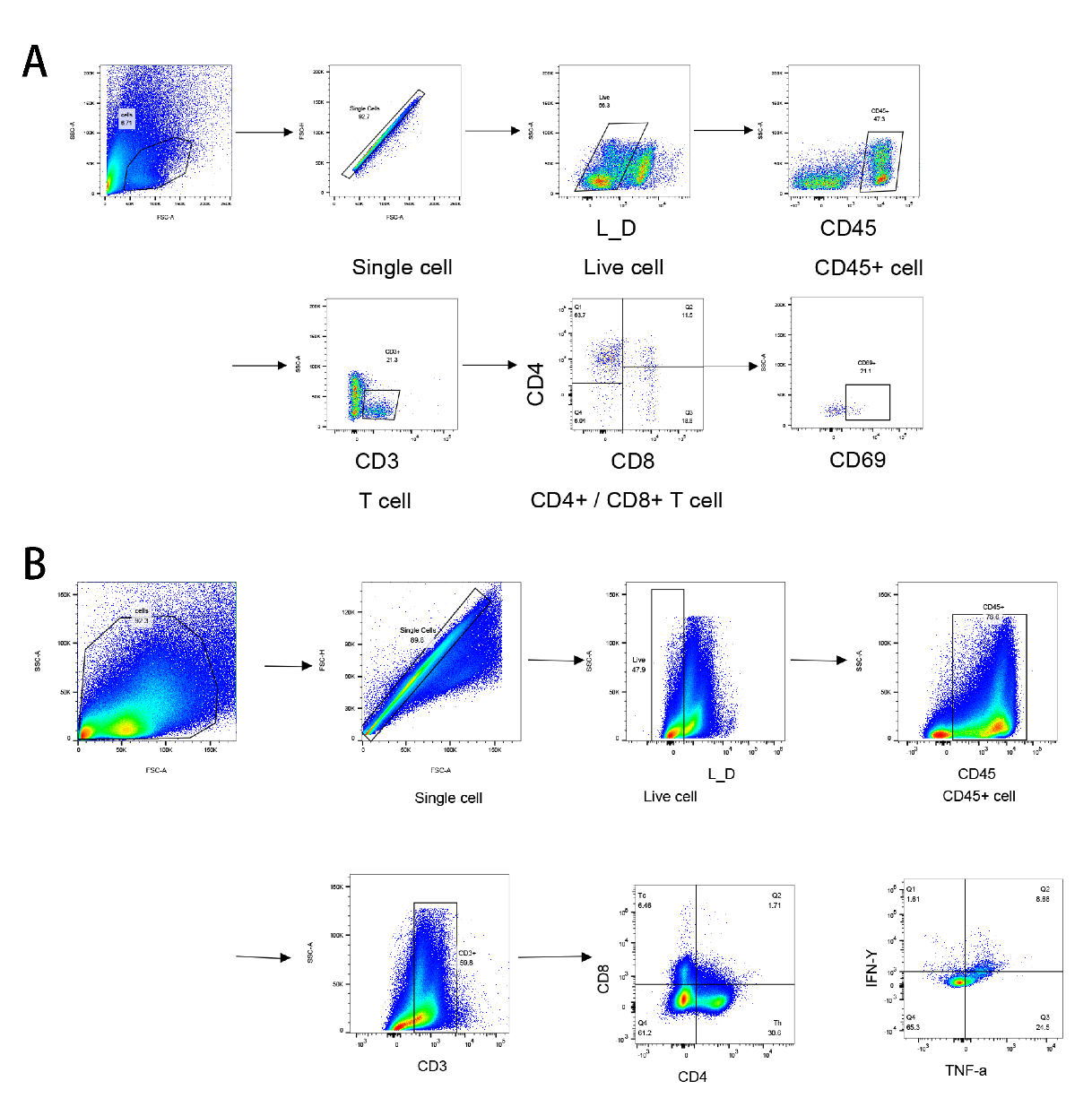
**

**Figure S4** (A) Gating information for flow cytometry of T cells in murine aortic and lymph-node samples. (B) Gating information for flow cytometry of T cells in murine spleen samples.

**
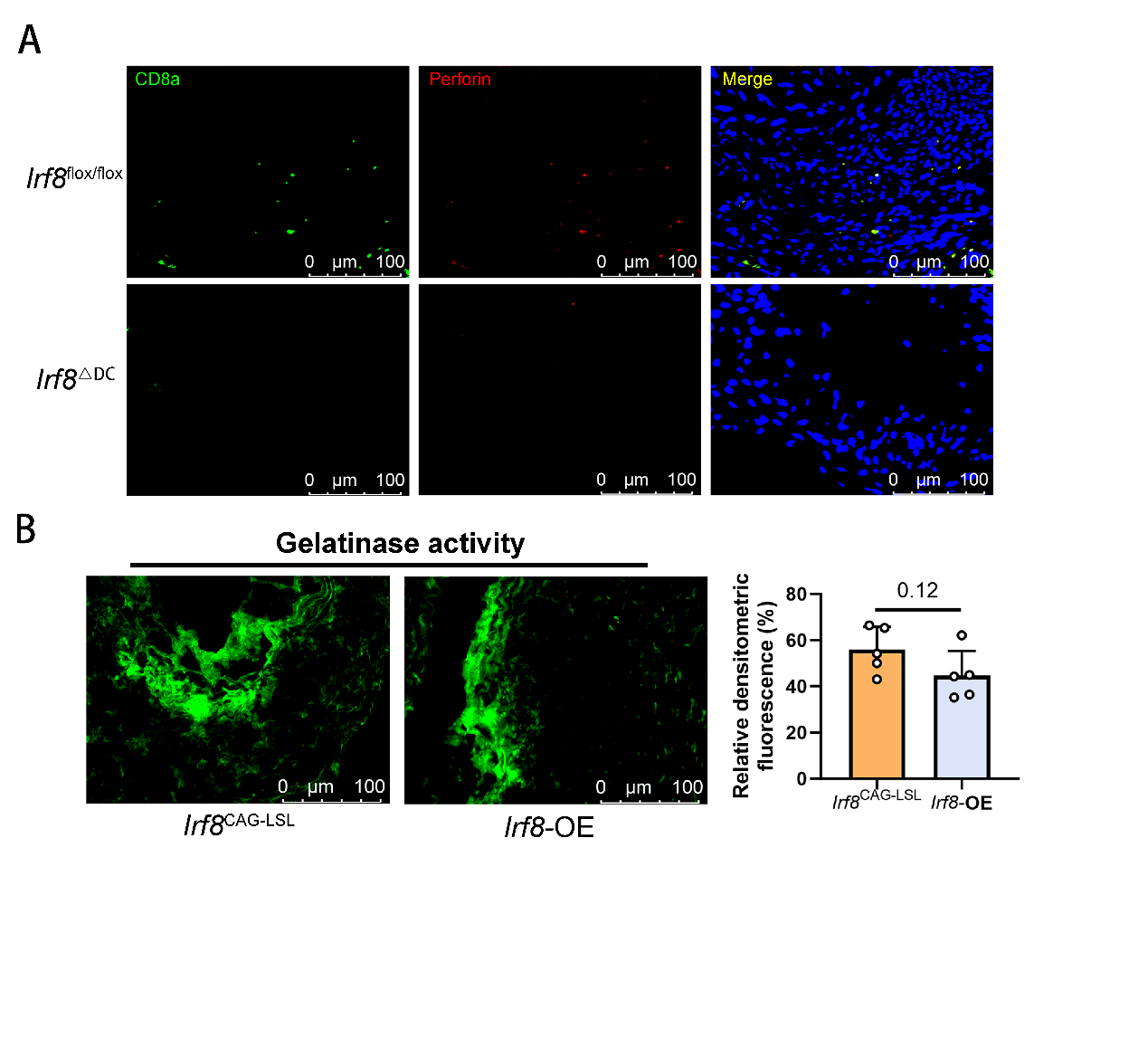
**

**Figure S5** (A) Representative immunofluorescence images of control (*Irf8*^flox/flox^) and *Irf8*^△DC^ aortic sections after peri-adventitial elastase application with staining for CD8a, perforin and nuclei (n = 3 of each group). Scale bar, 100 μm. (B) Representative *in situ*-zymography images of control and *Irf8*-OE group aortic sections after peri-adventitial elastase application. Scale bar, 100 μm.

**
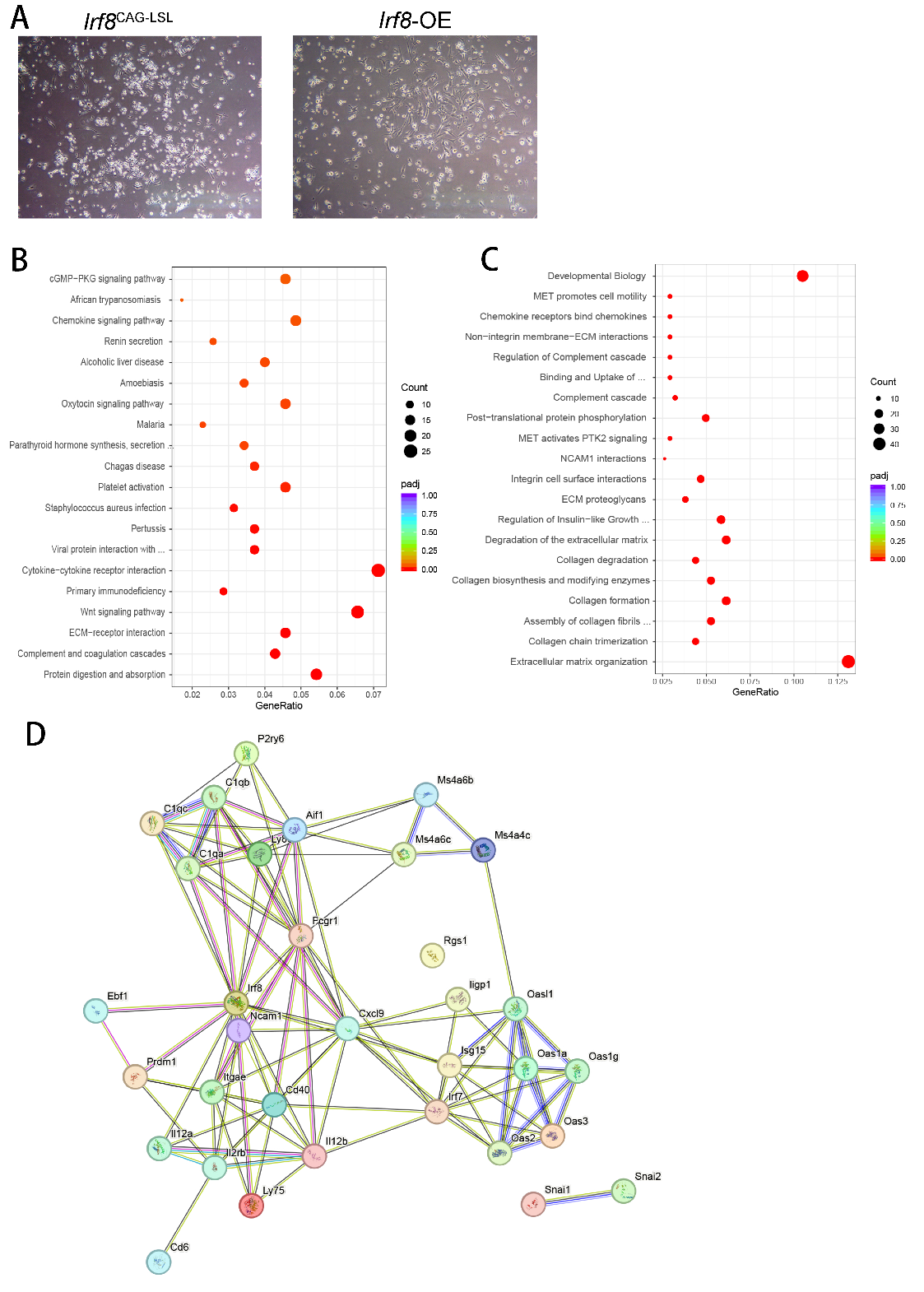
**

**Figure S6** (A) The morphology of the BMDCs from control (*Irf8*^CAG-LSL^) and *Irf8*-OE mice treated with GM-CSF plus Flt3l was observed under a light microscope (magnification: ×100). (B) Dot plot of KEGG enrichment analyses with terms of interest for the transcriptional level of upregulated genes of *Irf8*-OE BMDCs versus control ones. (*C*) Dot plot of Reactome enrichment analyses with terms of interest for the transcriptional level of upregulated genes of IRF8-OE BMDCs versus control ones. (*D*) Protein-protein interaction networks of DEGs in OE BMDCs subgroup related to IRF8 according to STRING database.

**
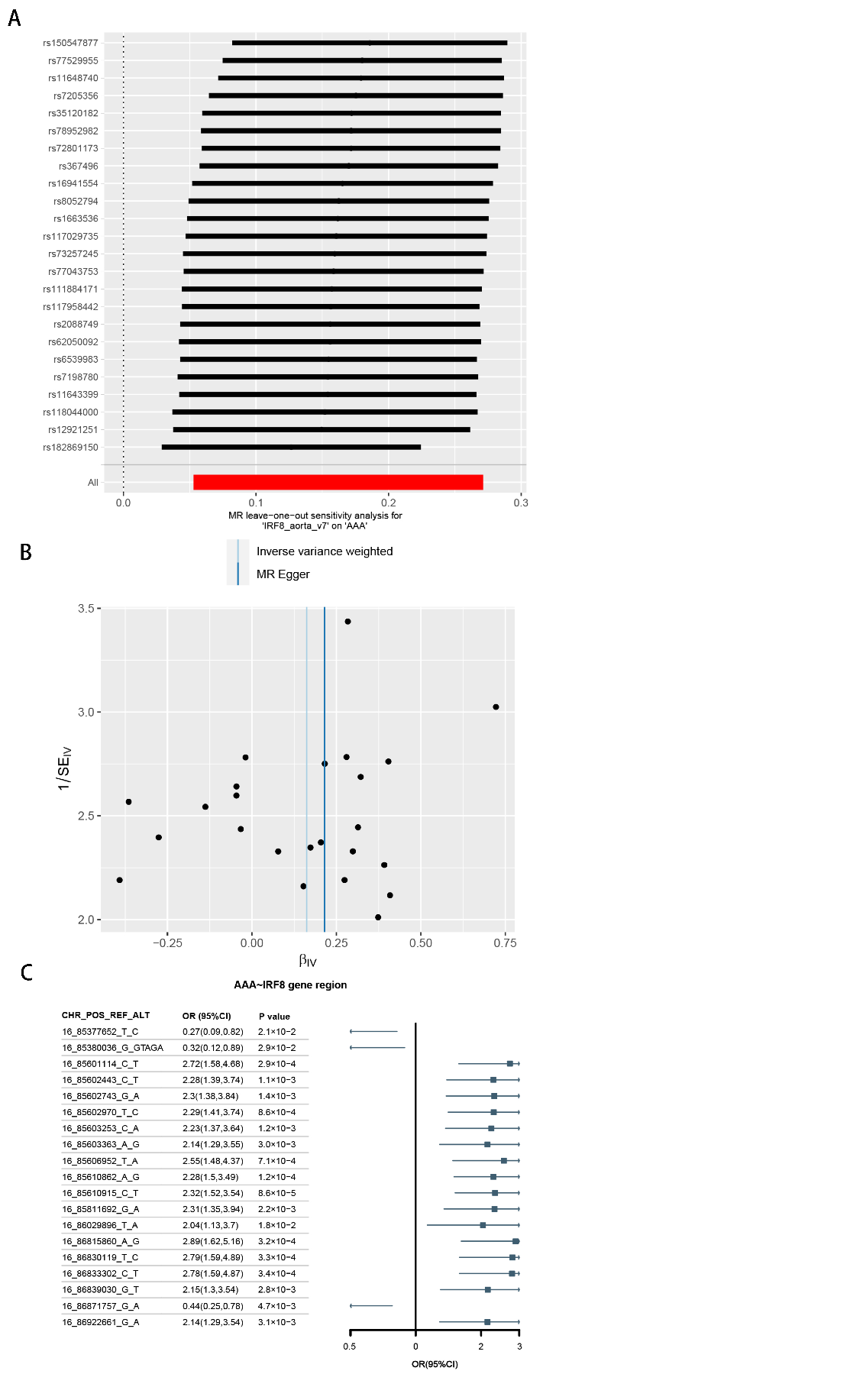
**

**Figure S7** (A) MR leave−one−out sensitivity analysis (n = 24). (B) Funnel plot between IRF8 eQTLs and AAA (n = 24). (C) Forest plot exhibiting the effect of specific SNPs of IRF8 on AAA development.

**Supplemental Table 1. MR results of Granzyme B pQTL and AAA**

| **id.exposure** | **id.outcome** | **method** | **nsnp** | **b** | **se** | **pval** |
| --- | --- | --- | --- | --- | --- | --- |
| Granzyme B | AAA | Maximum likelihood | 89 | 0.044759 | 0.01959 | 0.022322 |
| Granzyme B | AAA | MR Egger | 89 | 0.082869 | 0.033218 | 0.014496 |
| Granzyme B | AAA | MR Egger (bootstrap) | 89 | 0.06379 | 0.032032 | 0.022 |
| Granzyme B | AAA | Simple median | 89 | 0.050648 | 0.03129 | 0.105514 |
| Granzyme B | AAA | Weighted median | 89 | 0.065953 | 0.031396 | 0.035667 |
| Granzyme B | AAA | Penalised weighted median | 89 | 0.065951 | 0.030334 | 0.029695 |
| Granzyme B | AAA | Inverse variance weighted | 89 | 0.044542 | 0.019473 | 0.022176 |
| Granzyme B | AAA | IVW radial | 89 | 0.044544 | 0.017633 | 0.011532 |
| Granzyme B | AAA | IVW (multiplicative random effects) | 89 | 0.044542 | 0.017634 | 0.011538 |
| Granzyme B | AAA | IVW (fixed effects) | 89 | 0.044542 | 0.019473 | 0.022176 |
| Granzyme B | AAA | Simple mode | 89 | 0.103088 | 0.051297 | 0.047533 |
| Granzyme B | AAA | Weighted mode | 89 | 0.072003 | 0.029053 | 0.015106 |

**Supplemental Table 2. Baseline heart rate and systolic blood pressure**

|  | **Heart rate (beats/min, Mean ± SD ,n=8)** | **Systolic pressure (mmHg,**  **Mean ± SD, n=8)** |
| --- | --- | --- |
| *Irf8*^CAG-LSL^ | 602.13 ± 26.43 | 110.88 ± 7.55 |
| *Irf8*-OE | 597.00 ± 65.01 | 108.50 ± 4.60 |
| *Irf8*^flox/flox^ | 585.63 ± 22.43 | 107.63 ± 7.25 |
| *Irf8*^ΔDC^ | 603.25 ± 35.33 | 107.25 ± 6.43 |
| *Batf3*^-/-^ | 600.25 ± 62.85 | 112.63 ± 5.83 |
| *Clec9a*^-/-^ | 596.50 ± 59.92 | 108.50 ± 4.96 |
| WT | 591.50 ± 45.11 | 111.38 ± 5.42 |

**Supplemental Table 3. Representative reports of complete blood count**

|  | *Irf8*^CAG-LSL^ | *Irf8*-OE | *Irf8*^flox/flox^ | *Irf8*^ΔDC^ | *Batf3*^-/-^ | *Clec9a*^-/-^ | WT | **Units** | **Reference Range** |
| --- | --- | --- | --- | --- | --- | --- | --- | --- | --- |
| **White blood cell count** | 11.6 | 12.2 | 7.8 | 9 | 8.7 | 7.9 | 7.4 | **10^9/L** | 0.8-10.6 |
| **Lymphocyte count** | 7.6 | 7.9 | 5.6 | 5.9 | 6.5 | 5.7 | 5.5 | **10^9/L** | 0.6-8.9 |
| **Monocyte count** | 0.4 | 0.4 | 0.2 | 0.3 | 0.2 | 0.1 | 0.2 | **10^9/L** | 0.04-1.4 |
| **Neutrophil count** | 3.6 | 3.9 | 2 | 2.8 | 2 | 2.1 | 1.7 | **10^9/L** | 0.23-3.6 |
| **Lymphocyte percentage** | 65.5 | 64.7 | 71 | 65.4 | 74.7 | 71.7 | 74.2 | **%** | 40-92 |
| **Monocyte percentage** | 3.1 | 3.3 | 2.9 | 3.8 | 2.1 | 2.3 | 2.7 | **%** | 0.9-18 |
| **Neutrophil percentage** | 31.4 | 32 | 26.1 | 30.8 | 23.2 | 26 | 23.1 | **%** | 6.5-50 |
| **Red blood cell count** | 9.29 | 8.82 | 9.02 | 9.73 | 8.75 | 9.43 | 9 | **10^12/L** | 6.5-11.5 |
| **Hemoglobin** | 147 | 144 | 146 | 161 | 147 | 153 | 143 | **g/L** | 110-165 |
| **Hematocrit** | 47.9 | 44.8 | 45.3 | 47.7 | 45.6 | 47.9 | 43.6 | **%** | 35-55 |
| **Mean corpuscular volume (MCV)** | 51.6 | 50.9 | 50.3 | 49.1 | 52.2 | 50.9 | 48.5 | **fL** | 41-55 |
| **Mean corpuscular hemoglobin (MCH)** | 15.8 | 16.3 | 16.1 | 16.5 | 16.8 | 16.2 | 15.8 | **pg** | 13-18 |
| **Mean corpuscular hemoglobin**  **concentration (MCHC)** | 306 | 321 | 322 | 337 | 322 | 319 | 327 | **g/L** | 300-360 |
| **Red cell distribution width (RDW)** | 15.5 | 16.1 | 15.8 | 17.9 | 15.8 | 14.9 | 15.1 | **%** | 12月19日 |
| **Platelet count** | 1264 | 1158 | 928 | 793 | 1635 | 1534 | 1668 | **10^9/L** | 400-1600 |
| **Mean platelet volume (MPV)** | 6.4 | 6.4 | 6.6 | 5.3 | 6.4 | 6.3 | 6 | **fL** | 4.0-6.2 |
| **Platelet distribution width (PDW)** | 16.5 | 16.4 | 16.8 | 16.1 | 16.3 | 16.3 | 16.1 |  | 12.0-17.5 |

| Group | Final measurement | Initial measurement | Group | Final measurement | Initial measurement |
| --- | --- | --- | --- | --- | --- |
| *Irf8*^CAG-LSL^ | 0.92 | 0.4 | *Batf3*^-/-^ | 0.64 | 0.52 |
| *Irf8*^CAG-LSL^ | 1.12 | 0.52 | *Batf3*^-/-^ | 0.88 | 0.58 |
| *Irf8*^CAG-LSL^ | 0.9 | 0.48 | *Batf3*^-/-^ | 1.02 | 0.64 |
| *Irf8*^CAG-LSL^ | 1.06 | 0.6 | *Batf3*^-/-^ | 0.72 | 0.46 |
| *Irf8*^CAG-LSL^ | 1.2 | 0.6 | *Batf3*^-/-^ | 0.56 | 0.4 |
| *Irf8*^CAG-LSL^ | 1.14 | 0.58 | *Batf3*^-/-^ | 0.9 | 0.6 |
| *Irf8*^CAG-LSL^ | 0.7 | 0.36 | Wild type | 0.88 | 0.42 |
| *Irf8*^CAG-LSL^ | 1.3 | 0.58 | Wild type | 1.2 | 0.6 |
| *Irf8*-OE | 1.1 | 0.42 | Wild type | 0.98 | 0.52 |
| *Irf8*-OE | 1.32 | 0.52 | Wild type | 1.22 | 0.54 |
| *Irf8-OE* | 1.12 | 0.46 | Wild type | 0.94 | 0.56 |
| *Irf8-OE* | 1.3 | 0.58 | *Clec9a*^-/-^ | 0.62 | 0.44 |
| *Irf8-OE* | 1.4 | 0.54 | *Clec9a*^-/-^ | 0.76 | 0.56 |
| *Irf8-OE* | 1.32 | 0.56 | *Clec9a*^-/-^ | 0.5 | 0.44 |
| *Irf8-OE* | 1.28 | 0.48 | *Clec9a*^-/-^ | 0.84 | 0.6 |
| *Irf8-OE* | 1.18 | 0.42 | *Clec9a*^-/-^ | 0.5 | 0.38 |
| *Irf8*^flox/flox^ | 1.56 | 0.82 | IgG | 1.2 | 0.48 |
| *Irf8*^flox/flox^ | 0.92 | 0.52 | IgG | 0.94 | 0.5 |
| *Irf8*^flox/flox^ | 0.88 | 0.42 | IgG | 1.32 | 0.66 |
| *Irf8*^flox/flox^ | 1.24 | 0.6 | IgG | 1.14 | 0.52 |
| *Irf8*^flox/flox^ | 1.44 | 0.64 | IgG | 0.88 | 0.42 |
| *Irf8*^flox/flox^ | 0.98 | 0.52 | IgG | 1.08 | 0.6 |
| *Irf8*^ΔDC^ | 0.82 | 0.52 | CLEC9A Ab | 0.64 | 0.4 |
| *Irf8*^ΔDC^ | 0.76 | 0.46 | CLEC9A Ab | 0.58 | 0.4 |
| *Irf8*^ΔDC^ | 0.84 | 0.44 | CLEC9A Ab | 0.96 | 0.5 |
| *Irf8*^ΔDC^ | 0.68 | 0.44 | CLEC9A Ab | 0.54 | 0.42 |
| *Irf8*^ΔDC^ | 0.62 | 0.42 | CLEC9A Ab | 1.04 | 0.48 |
| *Irf8*^ΔDC^ | 0.88 | 0.5 | CLEC9A Ab | 0.58 | 0.44 |
| Wild type | 1.24 | 0.66 |  |  |  |
| Wild type | 1.3 | 0.58 |  |  |  |
| Wild type | 0.98 | 0.56 |  |  |  |
| Wild type | 0.88 | 0.4 |  |  |  |
| Wild type | 1.26 | 0.52 |  |  |  |
| Wild type | 1.14 | 0.7 |  |  |  |

**Supplemental Table 4. Measurements of aortic diameters (cm)**

**Major Resources Table**

In order to allow validation and replication of experiments, all essential research materials listed in the Methods should be included in the Major Resources Table below. Authors are encouraged to use public repositories for protocols, data, code, and other materials and provide persistent identifiers and/or links to repositories when available. Authors may add or delete rows as needed.

**Animals (in vivo studies)**

| **Species** | **Vendor or Source** | **Background Strain** | **Sex** | **Persistent ID / URL** |
| --- | --- | --- | --- | --- |
| Mus musculus | Slac Laboratory Animal Co. Ltd | C57BL/6 | Male | IMSR_JAX:000664 |
| ApoE KO | Slac Laboratory Animal Co. Ltd | C57BL/6 x 129 | Male | IMSR_JAX:002052 |
| *Irf8*^CAG-LSL^*Itgax*^Cre^ | GemPharmatech Co., Ltd | C57BL/6 | Male |  |
| *Irf8*^flox/flox^*Itgax*^Cre^ | GemPharmatech Co., Ltd | C57BL/6 | Male |  |
| *Batf3*^-/-^ | GemPharmatech Co., Ltd | C57BL/6 | Male |  |
| *Clec9a*^-/-^ | GemPharmatech Co., Ltd | C57BL/6 | Male |  |

**Antibodies**

| **Target antigen** | **Vendor or Source** | **Catalog #** | **Working concentration** | **Persistent ID / URL** |
| --- | --- | --- | --- | --- |
| MHC II | Invitrogen | 14-5321-82 | 2.5 μg/ml | https://www.thermofisher.com/antibody/product/14-5321-82.html?CID=AFLCA-14-5321-82 |
| HLA-DR | Bio-Rad Laboratories | MCA71R | 5 μg/ml | https://www.bio-rad-antibodies.com/monoclonal/human-hla-dr-antibody-ye2-36-hlk-mca71.html?utm_source=citeab.com&utm_medium=referral&utm_campaign=3rd+party+directory |
| Donkey Anti-Mouse | Abcam | ab150105 | 5 µg/ml | https://www.abcam.com/products/secondary-antibodies/donkey-mouse-igg-hl-alexa-fluor-488-ab150105.html |
| Donkey Anti-Rat | Thermo | A-21208 | 5 µg/ml | https://www.thermofisher.cn/cn/zh/antibody/product/Donkey-anti-Rat-IgG-H-L-Highly-Cross-Adsorbed-Secondary-Antibody-Polyclonal/A-21208 |
| Goat Anti-Rat | Thermo | A-21247 | 5 µg/ml | https://www.thermofisher.com/antibody/product/A-21247.html?CID=AFLCA-A-21247 |
| Donkey Anti-Rabbit | Thermo | A10042 | 5 µg/ml | https://www.thermofisher.cn/cn/zh/antibody/product/Donkey-anti-Rabbit-IgG-H-L-Highly-Cross-Adsorbed-Secondary-Antibody-Polyclonal/A10042?adobe_mc=MCMID%7C80338808300360087390628211476740511377%7CMCAID%3D32C546D54FD5BB94-40000FAB22D91B49%7CMCORGID%3D5B135A0C5370E6B40A490D44%40AdobeOrg%7CTS=1614293705 |
| CD45 | BD Pharmingen | 557659 | 10 µg/ml | https://www.bdbiosciences.com/us/applications/research/stem-cell-research/cancer-research/mouse/apc-cy7-rat-anti-mouse-cd45-30-f11/p/557659 |
| CD3 | BD Pharmingen | 561798 | 10 µg/ml | https://www.bdbiosciences.com/us/applications/research/t-cell-immunology/th-1-cells/surface-markers/mouse/fitc-rat-anti-mouse-cd3-molecular-complex-17a2/p/561798 |
| CD19 | BD Pharmingen | 557398 | 10 µg/ml | https://www.bdbiosciences.com/us/applications/research/stem-cell-research/hematopoietic-stem-cell-markers/mouse/negative-markers/fitc-rat-anti-mouse-cd19-1d3/p/557398 |
| CD49b/NK1.1 | BD Pharmingen | 553857 | 10 µg/ml | https://www.bdbiosciences.com/us/reagents/research/antibodies-buffers/immunology-reagents/anti-mouse-antibodies/cell-surface-antigens/fitc-rat-anti-mouse-cd49b-dx5/p/553857 |
| Ly6G | BD Pharmingen | 566435 | 10 µg/ml | https://www.bdbiosciences.com/us/reagents/research/antibodies-buffers/immunology-reagents/anti-mouse-antibodies/cell-surface-antigens/bb700-rat-anti-mouse-ly-6g-1a8/p/566435 |
| CD11c | BD Pharmingen | 558079 | 10 µg/ml | https://www.bdbiosciences.com/us/reagents/research/antibodies-buffers/immunology-reagents/anti-mouse-antibodies/cell-surface-antigens/pe-cy7-hamster-anti-mouse-cd11c-hl3/p/558079 |
| MHC II | BD Pharmingen | 742893 | 10 µg/ml | https://www.bdbiosciences.com/us/reagents/research/antibodies-buffers/immunology-reagents/anti-mouse-antibodies/cell-surface-antigens/bv510-rat-anti-mouse-i-ai-e-m5114152-also-known-as-m5114/p/742893 |
| CD64 | BD Pharmingen | 558539 | 10 µg/ml | https://www.bdbiosciences.com/us/applications/research/stem-cell-research/cancer-research/mouse/alexa-fluor-647-mouse-anti-mouse-cd64-a-and-b-alloantigens-x54-571/p/558539 |
| F4/80 | BD Pharmingen | 565411 | 10 µg/ml | https://www.bdbiosciences.com/us/reagents/research/antibodies-buffers/immunology-reagents/anti-mouse-antibodies/cell-surface-antigens/bv421-rat-anti-mouse-f480-t45-2342/p/565411 |
| CD11b | BD Pharmingen | 740861 | 10 µg/ml | https://www.bdbiosciences.com/en-us/products/reagents/flow-cytometry-reagents/research-reagents/single-color-antibodies-ruo/bv786-rat-anti-cd11b.740861 |
| CD103 | BD Pharmingen | 557495 | 10 µg/ml | https://www.bdbiosciences.com/en-us/products/reagents/flow-cytometry-reagents/research-reagents/single-color-antibodies-ruo/pe-rat-anti-mouse-cd103.557495 |
| L/D | BD Pharmingen | 565694 | 10 µg/ml | https://www.bdbiosciences.com/en-br/products/reagents/flow-cytometry-reagents/research-reagents/single-color-antibodies-ruo/fixable-viability-stain-575v.565694 |
| CD3 | BD Pharmingen | 740147 | 10 µg/ml | https://www.bdbiosciences.com/en-us/products/reagents/flow-cytometry-reagents/research-reagents/single-color-antibodies-ruo/bv510-rat-anti-mouse-cd3-molecular-complex.740147 |
| CD4 | BD Pharmingen | 552775 | 10 µg/ml | https://www.bdbiosciences.com/en-us/products/reagents/flow-cytometry-reagents/research-reagents/single-color-antibodies-ruo/pe-cy-7-rat-anti-mouse-cd4.552775 |
| CD69 | BD Pharmingen | 560689 | 10 µg/ml | https://www.bdbiosciences.com/en-ca/products/reagents/flow-cytometry-reagents/research-reagents/single-color-antibodies-ruo/apc-hamster-anti-mouse-cd69.560689 |
| CD11c | CST | #45581 | 0.8 µg/ml | https://www.cellsignal.com/products/primary-antibodies/cd11c-d3v1e-xp-rabbit-mab/45581 |
| IRF8 | Invitrogen | 17-9852-82 | 2.5 µg/ml | https://www.thermofisher.cn/cn/zh/antibody/product/IRF8-Antibody-clone-V3GYWCH-Monoclonal/17-9852-82 |
| TNFα | Fisher | 2282681 | 10 µg/ml | https://www.fishersci.com/shop/products/anti-tnf-clone-mp6-xt22-bd-3/BDB563387 |
| IFN-γ | Fisher | 554412 | 10 µg/ml | https://www.fishersci.com/shop/products/ifn-rat-anti-mouse-pe-clone-xmg1-2-bd/BDB554412 |
| Cleaved Caspase-3 | CST | #9661 | 1:400 | https://www.cellsignal.com/products/primary-antibodies/cleaved-caspase-3-asp175-antibody/9661 |
| Cathepsin K | Santa Cruz | sc-48353 | 2 µg/ml | https://www.scbt.com/p/cathepsin-k-antibody-e-7 |

**Other**

| **Description** | **Source / Repository** | **Persistent ID / URL** |
| --- | --- | --- |
| Elastase from porcine pancreas | Sigma-Aldrich | E1250 |
| Collagenase I | Gibco | 17100017 |
| Collagenase XI | Sigma-Aldrich | C7657 |
| Hyaluronidase | Sigma-Aldrich | H3506 |
| DNase I | Roche | 11284932001 |
| TSA 7-color kit | Absinbio | abs50015-100T |
| anti-rabbit/mouse HRP secondary antibody | Absinbio | #A10011-60 |
| EnzChek Gelatinase / Collagenase Assay Kit | Molecular Probe/ Invitrogen | E12055 |

Irf8 siRNA sequence: Sense strand (5'-3'): GGGACAACACCAUCUUCAA(dT)(dT)

Antisense strand (5'-3'): UUGAAGAUGGUGUUGUCCC(dT)(dT)
